# Not all infected fish become mushy: phenotypic expression underestimates *Unicapsula seriolae* infection in yellowtail kingfish (*Seriola lalandi*)

**DOI:** 10.1007/s00436-026-08735-w

**Published:** 2026-07-28

**Authors:** Jessica Tout-Lyon, Megan Porter, Christopher M. Whipps, Shokoofeh Shamsi

**Affiliations:** 1 Aquatic Molecular and Microbial Ecology Research (AMMbER) lab, School of Agricultural, Environmental and Veterinary Sciences, Faculty of Science and Health, and Gulbali Institute, Charles Sturt University, Birpai Country, Guruk (Port Macquarie), Australia; 2 SUNY ESF, Environmental Biology, State University of New York College of Environmental Science and Forestry, 1 Forestry Drive, Syracuse, NY 13210 USA; 3 School of Agricultural, Environmental and Veterinary Sciences, Faculty of Science and Health, and Gulbali Institute, Charles Sturt University, Wiradjuri Country, Wagga Wagga, Australia

**Keywords:** *Unicapsula*, Mushy flesh, Fisheries, Citizen-science, Parasite

## Abstract

Yellowtail Kingfish (YTK) (*Seriola lalandi*) is widely distributed throughout the Indo-Pacific and is a significant recreational and commercial species in Australia. In New South Wales (NSW), a post-cooking mushy flesh condition has been reported, though its cause remains unconfirmed. This study combined morpho-molecular diagnostics with a citizen-science mushy fish reporting platform to identify associated parasites and to map the spatio-temporal distribution of mushy flesh and infections in NSW. *Unicapsula seriolae* was detected in 38.9% (95% exact CI: 23.1–56.5%) of YTK donated by recreational anglers and charter operators (*n* = 36) (2024–2025) and confirmed by myxospore morphology and SSU and LSU rDNA sequencing. Most infected fish (71.4%) did not exhibit the phenotype, although mushy individuals were significantly more likely to be infected than non-mushy fish. Infection occurred year-round and across bioregions, whereas the phenotype was restricted to warmer months, yet asymptomatic infections were also present. Collectively, these findings extend the records of *U. seriolae*-YTK infections on Australia’s east coast beyond the initial reports off QLD and indicate that mushy flesh alone is an unreliable indicator of *U. seriolae* infection. Infection and sea surface temperature alone were insufficient to drive phenotypic expression, with host–environment–parasite interactions likely modulating its manifestation. Citizen-science data (*n* = 48) revealed widespread, temporally variable phenotypic occurrence and documented socio-economic impacts on anglers and consumers. The parasite’s effects on flesh quality may influence angler and consumer perception and marketability; however, the weak concordance between infection and phenotype indicates that visual assessment alone is likely to underestimate infection, allowing infected fish to enter the supply chain, undetected. Although *U. seriolae* is not considered a significant zoonotic parasite, in Japan, reports of foodborne illness associated with *U. seriolae* in other *Seriola* species suggest these findings warrant consideration from a food safety perspective. The results highlight that *U. seriolae* is present in YTK from the Manning and Hawkesbury shelf bioregions in NSW, but the expression of mushy flesh is conditional rather than a direct, inevitable consequence of infection.

**Supplementary Information:**

The online version contains supplementary material available at https://doi.org/10.1007/s00436-026-08735-w.

## Introduction

Yellowtail Kingfish (YTK) (*Seriola lalandi*) Valenciennes, 1833, belonging to the family Carangidae, is an important recreational and commercial gamefish species in Australia, also supporting aquaculture in South Australia (SA) (Chai et al. 2009; Paduano et al. 2026; Stone et al. 2022). YTK is a fast-growing pelagic dioecious carnivore that is distributed through the Indo-Pacific and is highly prized for sashimi (Froese and Pauly 2025; Liu et al. 2022; Symonds et al. 2014) with tail lengths reported up to 250 cm and bodyweights exceeding 96 kg (Bray 2025).

A key issue affecting some YTK landed in New South Wales (NSW) is the development of mushy flesh following cooking, rendering affected fish unpalatable and resulting in economic losses for the commercial sector and reduced value for recreational catches (FRDC, 2023; Kleindienst 2014; Lester 1982; Miller and Adlard 2013). Affected fish are organoleptically indistinguishable from unaffected individuals at landing and when prepared for raw consumption as sashimi. Appearance, smell, and taste remain similar until cooking, preventing the detection of the issue prior (Lester 1982) and confirmation of whether a YTK will develop mushy flesh therefore remains dependent on cooking. Although parasite-derived protease activity may potentially be the underlying mechanism (Samaranayaka et al. 2007; Tsuyuki 1982) for the development of mushy flesh in YTK on cooking (Miller 2024), it remains unverified.

Myxozoan parasites, including species of *Unicapsula* Davis, 1924, and *Kudoa* Meglitsch, 1947 are known to infect fish musculature and can cause post-mortem myoliquefaction (Miller and Adlard 2013; Tsuyuki 1982; Yunus et al. 2021). These parasites are globally distributed across marine and freshwater ecosystems and can significantly impact fisheries (Born-Torrijos et al. 2022; Holzer et al. 2021). While some of these parasites are often undetectable at landing (Li et al. 2022; Shamsi and Barton 2024), infection prevalences have increased markedly in some systems, with up to six-fold rises reported over short timeframes, leading to economic losses and, in some cases, food-borne illness (Giulietti et al. 2022a; Iwashita et al. 2013; Jinnai et al. 2017; Kodo et al. 2026; Ohnishi et al. 2016, 2018). In Japan, food-borne illnesses have been associated with the consumption of raw Greater Amberjack (*Seriola dumerili* Risso, 1810), a closely related species to YTK, infected with the myxosporean parasite *Unicapsula seriolae* Lester 1982 (Kodo et al. 2026; Ogawa 2023; Ohnishi et al. 2016, 2018). Although such illnesses have not been reported in Australia, projected global range shifts highlight the need to assess parasite presence and distribution in Australian waters (Shamsi and Barton 2024).

Previous reports of parasite infections in Australian YTK are important from a fisheries surveillance and seafood-quality perspective, as parasite occurrence in commercially and recreationally important fish species may influence marketability, consumer confidence, and perceptions of seafood safety, even where direct zoonotic risk is absent. Parasites including myxozoans, anisakid nematodes, and monogeneans have previously been reported from Australian YTK and incorporated into aquaculture biosecurity and parasite risk-assessment frameworks (Hutson et al. 2007a, b; Shamsi 2021; Shamsi et al. 2011). Qualitative risk assessments for Australian YTK aquaculture and elsewhere have identified parasites such as *U. seriolae* among those with the potential to negatively affect sustainability, marketability and profitability of sea-cage production systems (Bartholomew and Kerans 2015; Hutson 2007; Hutson et al. 2007a, b). Establishing baseline knowledge of parasite diversity, distribution, and associated phenotypic outcomes in Australian waters is therefore essential for understanding emerging host–parasite dynamics under changing environmental conditions and for informing evidence-based fisheries management, seafood-quality assurance, and public communication.

In Australia, myxozoan infections including *Kudoa*, *Ceratomyxa*, and *Unicapsula* spp. have been documented in both wild and cultured YTK across multiple regions (Hutson et al. 2007a, b; Lester 1982; Rohde 1976; Stephens and Savage 2010). The mushy flesh phenotype and infection with *U. seriolae* has been confirmed in wild YTK from Queensland (QLD) and in both wild and cultured fish from Western Australia (WA), across both genetic populations of the species (Kleindienst 2014; Lester 1982; Miller et al. 2011; Miller and Adlard 2013; Stephens and Savage 2010; Stephens 2016). In WA-based YTK aquaculture systems, *U. seriolae* infections were reported under a range of conditions from persistent background infections (Kleindienst 2014), to episodes of significant mortality (> 70% in YTK) and post-mortality events (Stephens and Savage 2010). Although multiple pathogens were present during these events and no single causative agent was identified, these observations suggest that myxozoan infections may contribute to disease outcomes under environmental or physiological stress in YTK (Stephens and Savage 2010). In contrast, despite the first reports of mushy flesh in NSW YTK in 1991 (Smith et al. 1991), the associated parasite(s) have not been confirmed, and Koch’s postulates (Hutson et al. 2023; Koch 1884) remain unfulfilled.

This uncertainty is compounded by methodological limitations in earlier NSW-based YTK studies, which relied on phenotypic observations or microscopy alone (Forrest 2019; Hutson et al. 2007a, b; Smith et al. 1991). While spores and plasmodia of *U. seriolae* can in some instances be visible to the naked eye (Lester 1982; Tomochi et al. 2014), they are not always macroscopic in infected fish (Ogawa 2023); further complicating diagnosis based on visual phenotype alone and driving organoleptic similarities between infected and non-infected fish. Consequently, the presence and distribution, prevalence, and environmental drivers of infection in NSW YTK are poorly understood. Ongoing ocean warming and predicted poleward shifts in YTK distribution (Champion et al. 2019; Phillips et al. 2022), raise the question of whether their associated parasites are experiencing similar range shifts. Changes in their presence, distribution, and phenotypic expression could affect fisheries sustainability and satisfaction and confidence among anglers and consumers.

This study aimed to investigate these knowledge gaps by integrating morpho-molecular diagnostics with citizen-science reporting to investigate the parasites associated with mushy flesh in NSW YTK, map their distribution, and evaluate their co-occurrence with mushy flesh. Specifically, the study aimed to: (1) identify the parasite species associated with mushy flesh in NSW YTK using morphological and molecular approaches; (2) map the spatio-temporal occurrence of mushy flesh and infections using both citizen-science reporting and molecular findings; and (3) compare infection prevalence with previous Australian studies.

## Materials and methods

A total of 36 wild-caught YTK were donated as tissue samples by recreational anglers and charter operators (including Wildside Sportfishing in South West Rocks and Fish About Sydney Harbour). Samples were collected during 2024 and into January and February 2025 from a variety of locations spanning ~ 365 km of coastline from Grassy Head in the north on the mid north coast of NSW (Manning shelf bioregion) to Sydney Harbour in the south of NSW (Hawkesbury shelf bioregion).

Fish tissue samples were deposited by anglers into either 5 mL DNA LoBind tubes (Eppendorf), 50 mL falcon tubes (Eppendorf), or zip-lock bags and placed into residential freezers until collection. Anglers donated trunk muscle collected from randomly selected lateral/longitudinal positions. To capture authentic real‑world recreational practices, anglers were not instructed to control or standardise post‑harvest practices among fish caught and instead were encouraged to continue their own harvest and intermediate storage techniques prior to freezing. The exact handling/storage histories were assumed heterogeneous and unrecorded. The date and location of the catch and fish details including length (cm), weight (kg) and whether the fish turned mushy on cooking, prepared as ceviche or when smoked, were recorded (reporting was not possible for all cases). Where fish length was reported as a range by anglers across multiple fish landed from the same event, the midpoint of the range was used for analysis. Water temperature data were obtained from anglers where possible and when these were unavailable, values were supplemented using publicly available sea surface temperature.

(SST) data (https://seatemperature.info/new-south-wales-water-temperature.html, matched to fishing location and date. Specimens were either collected in person or sent on ice to the Aquatic Molecular and Microbial Ecology Research (AMMbER) laboratory at Charles Sturt University (CSU) in Guruk (Port Macquarie), Birpai Country, NSW. Fish tissue samples were stored at -80 °C until further processing.

### Morpho-molecular analysis

Less than 30 mg of tissue sample from the trunk muscle of each fish was transferred into a 1.5 ml DNase and RNase free DNA LoBind tubes (Eppendorf). Genomic DNA was extracted using the E.Z.N.A Tissue DNA Kit (Omega Bio-Tek) according to the manufacturer’s guidelines and eluted in 200 µl of elution buffer. PCR amplification targeting part of the SSU rDNA gene was carried out using the forward primer Kud6F 5′- TCACTATCGGAATGAACG − 3′ (Whipps et al. 2003) and reverse primer 18R 5′- CTACGGAAACCTTGTTACG − 3′ (Whipps et al. 2003). Each 50 ul amplification reaction prepared with nuclease free water contained 3 µl template DNA, 1X One*Taq* Hot Start Quick-Load 2X Master Mix with Standard Buffer (New England BioLabs Inc.), 0.2 µM each primer. The PCR cycling conditions had the following steps: Initial denaturation at 95°C for 3 min, followed by 35 cycles of 94°C for 30 sec, 53°C for 45 sec, and 72°C for 90 sec. Cycling was followed by a final extension at 68°C for 7 min. An aliquot (2.5 µl) of each PCR reaction was examined on a 1.5% w/v agarose gel, stained with SYBR™ Safe and photographed upon transillumination. For large subunit rDNA (LSU rDNA) sequencing, the 5’ region containing variable regions D1 and D2, was targeted with primers Myxo28S2F (Whipps et al. 2021) and 28S1R (Whipps et al. 2004). PCR conditions were identical to those above except for an annealing temperature of 54 °C instead of 53 °C. Amplified products were purified using the E.Z.N.A. Cycle Pure Kit (Omega Bio-Tek, Norcross, Georgia). Direct sequencing of SSU and LSU was performed using the forward and reverse primers above.

In Australia, PCR purification and Sanger sequencing was conducted at the Australian Genome Research Facility (AGRF). Forward AB1 trace files (chromatograms) were visually inspected using Geneious Prime version 2024.05. A subset of tissue sample from each fish was sent in 15 mL falcon tubes on dry ice to State University New York (SUNY), Syracuse, United States of America for morpho-molecular analysis. Here, sequencing reactions were carried out with the ABI BigDye Terminator Cycle Sequencing Ready Reaction Kit v3.1, using the ABI3730xl Genetic Analyzer (Applied Biosystems, Foster City, California) at the W.M. Keck Foundation Biotechnology Resource Laboratory at Yale University. Reads were evaluated, and if applicable contigs were created, in BioEdit.

A maximum likelihood phylogenetic analysis was carried out on aligned sequences in PhyML (Guindon et al., 2010) with nucleotide substitution model (HKY85 + G + I) determined with Smart Model Selection (Lefort et al., 2017). Bootstrap values were calculated with 1,000 replicates. Phylogenetic trees were edited and annotated with Adobe Illustrator (Adobe Systems Inc., San Jose, California).

A subset of these tissues (*n* = 10) that were PCR-positive, were prepared for morphological examination following the method described by St. Hilaire et al. (1997) at SUNY ESF. Briefly, approximately 0.5 g of muscle tissue was minced in saline and then pressed between 2 acrylic plates. The fluid was collected in a 15 ml conical tube and allowed to settle before taking a droplet of material from the bottom of the tube to prepare a wet mount with a 50 mm coverslip. Slides were examined in their entirety using a Nikon 80i compound microscope and phase contrast at 200X magnification. Representative myxospores encountered were observed at 400X and 1000X magnification and photographed with a digital microscope camera.

### Spatiotemporal mapping of *U. seriolae* infection and mushy flesh phenotype occurrence

For all fish tissue samples donated and subjected to molecular analysis, the location of fish landings was mapped using ArcGIS Pro version 3.6.0. For locations of catches logged by anglers, an ESRI Survey123 application was developed to collect angler data, with submissions displayed in real time through an embedded ESRI Story Map (Supplementary Table 1). The platform was launched publicly in October 2024, allowing for both recreational and commercial anglers, and consumers to log their catch: https://storymaps.arcgis.com/stories/45c3bb3be5d146728566012f19012c9c. The data analysed here spans from October 2024 to November 2025.

Comments were anonymised, cleaned and analysed using a pragmatic thematic approach. Deductive codes (e.g., seasonality – when comments mention months/warmer water) were informed by mapping objectives, while inductive codes (e.g., frequency: when comments referred to timeframes such as “over the last 5 years” or “this season”; market/auction observation: when comments mention fish bought or sold) were generated through line-by-line reading. Themes were consolidated into several higher-level categories, and two representative anonymised quotes per theme were selected to illustrate angler observations and impacts.

### Comparison of *U. seriolae* infection prevalence with previous Australian studies

Previous data documenting the infection prevalence of *U. seriolae* in YTK landed in Australia by Lester (1982), Stephens and Savage (2010), Miller and Adlard (2013), and Kleindienst (2014) were collated for descriptive comparisons to our study. Exact binomial 95% confidence intervals were calculated using the Clopper–Pearson method (binom.test in RStudio (R version 4.5.2) and plotted. Fish landed were partitioned according to whether they were wild landings or from aquaculture settings.

### Statistical analysis

All statistical analyses were conducted in RStudio (R version 4.5.2). Associations between categorical variables and binary PCR detection outcomes were initially explored using Fisher’s Exact Tests (Supplementary Table S2). Descriptive analyses and univariable models were based on all 36 fish.

To explore multivariable relationships, binomial generalized linear models (logistic regression) were first fitted including candidate predictors (bioregion, water temperature, fish length, and mushy flesh phenotype). Model diagnostics indicated issues of small-sample bias and separation, evidenced by inflated standard errors and non-finite parameter estimates, particularly for categorical predictors.

To address these limitations, Firth penalised logistic regression was implemented using the *logistf* package (Heinze et al. 2025; Puhr et al. 2017), providing bias-reduced and finite estimates. Primary inference was based on Firth logistic regression models fitted to complete cases (*n* = 33).

A univariable Firth penalised logistic regression model was used to estimate the association between PCR detection and mushy flesh phenotype as a single-predictor relationship, as standard logistic regression exhibited separation and produced unstable estimates. This provided a bias-reduced estimate of this key bivariate association.

Given strong imbalance in seasonal sampling and separation issues, season was excluded from multivariable modelling due to sparse category counts (including a single observation in autumn) and associated instability in parameter estimation.

Multivariable models were specified to estimate adjusted associations between PCR detection and bioregion (categorical), water temperature (continuous, per 1 °C increase), fish length (continuous, per 1 cm increase), and mushy flesh phenotype (binary). Season was therefore not included in inferential modelling.

Adjusted predicted probabilities were derived from the multivariable Firth models, with continuous covariates held at their sample means and categorical variables set to reference levels. Nonparametric bootstrap resampling (1,000 iterations) was used to generate 95% confidence intervals for predicted probabilities.

Raw sample sizes for each predictor category and bioregion × season combination were reported alongside model outputs to aid interpretation under conditions of sparse and unbalanced sampling.

## Results

### DNA sequence confirmation

**Fig. 1:**
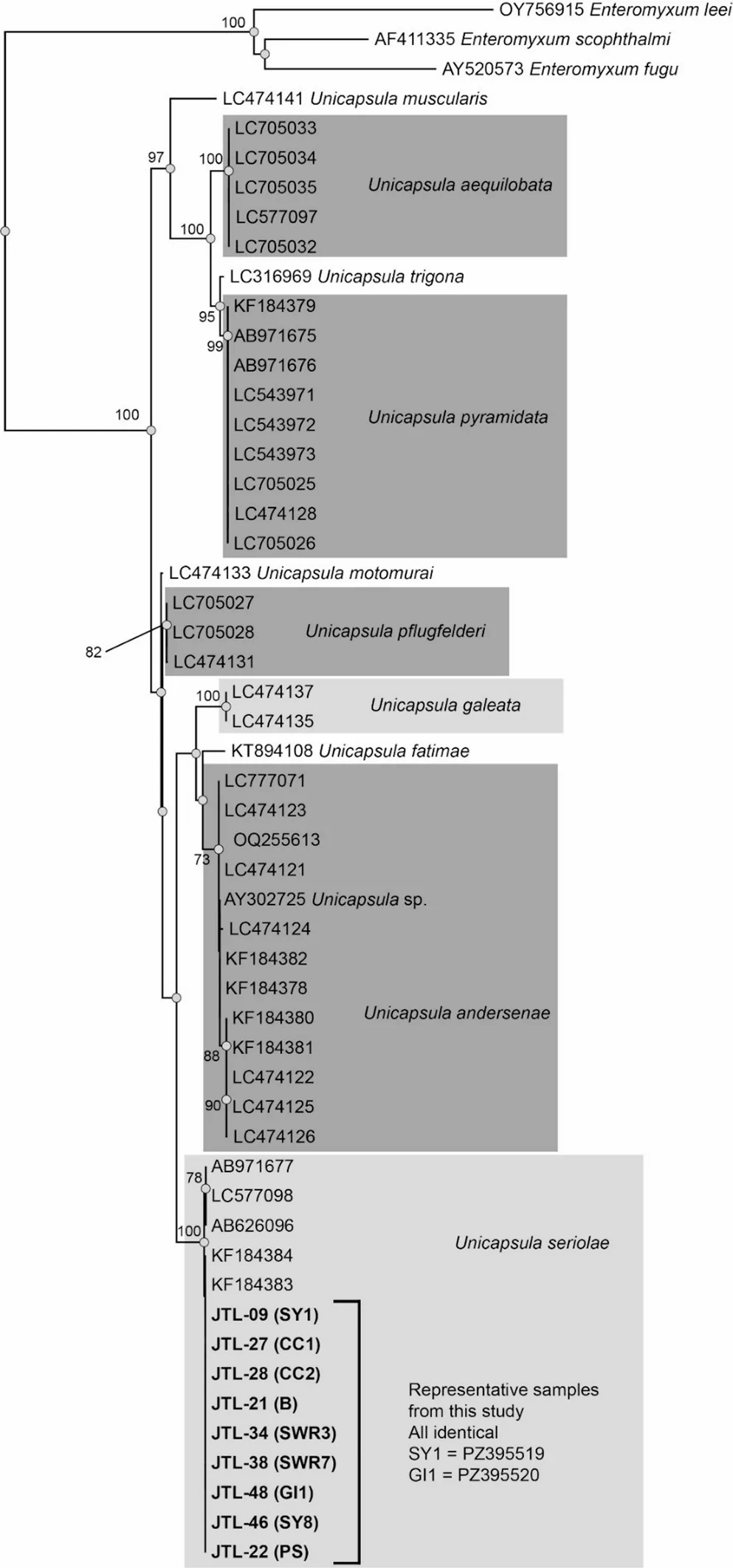
Phylogenetic tree from maximum likelihood analysis of small subunit ribosomal DNA sequences for *Unicapsula* species from Yellowtail Kingfish (*Seriola lalandi*). Sequences from samples collected in current study in bold. GenBank accession numbers are listed adjacent to species names. Support values in percent units at branching points, values under 65% not shown

The DNA sequences obtained from positive samples (Supplementary Table S3) were subjected to a GenBank BLAST search and identified as *U. seriolae*. Phylogenetically, sequences initially identified as belonging to *U. seriolae* from YTK clustered together and with other *U. seriolae* sequences from GenBank with high bootstrap support (Fig. 1). The sequences we obtained ranged in length from 800 to 980 nucleotides, and in overlapping regions were identical to one another and nearly identical to other *U. seriolae* sequences (e.g., AB971677). Two representative sequences were deposited in GenBank (PZ395519-PZ395520). Partial LSU rDNA sequences were also obtained from 15 specimens. Along their 680-nucleotide length, these sequences were 99% similar to one another, with 4 variable positions containing substitutions or ambiguous bases. Two sequences representative of this variation were deposited in GenBank (PZ395632-PZ395633). These were also 99% similar to existing *U. seriolae* sequences in GenBank (e.g., LC577099).

### Morphology

**Fig. 2:**
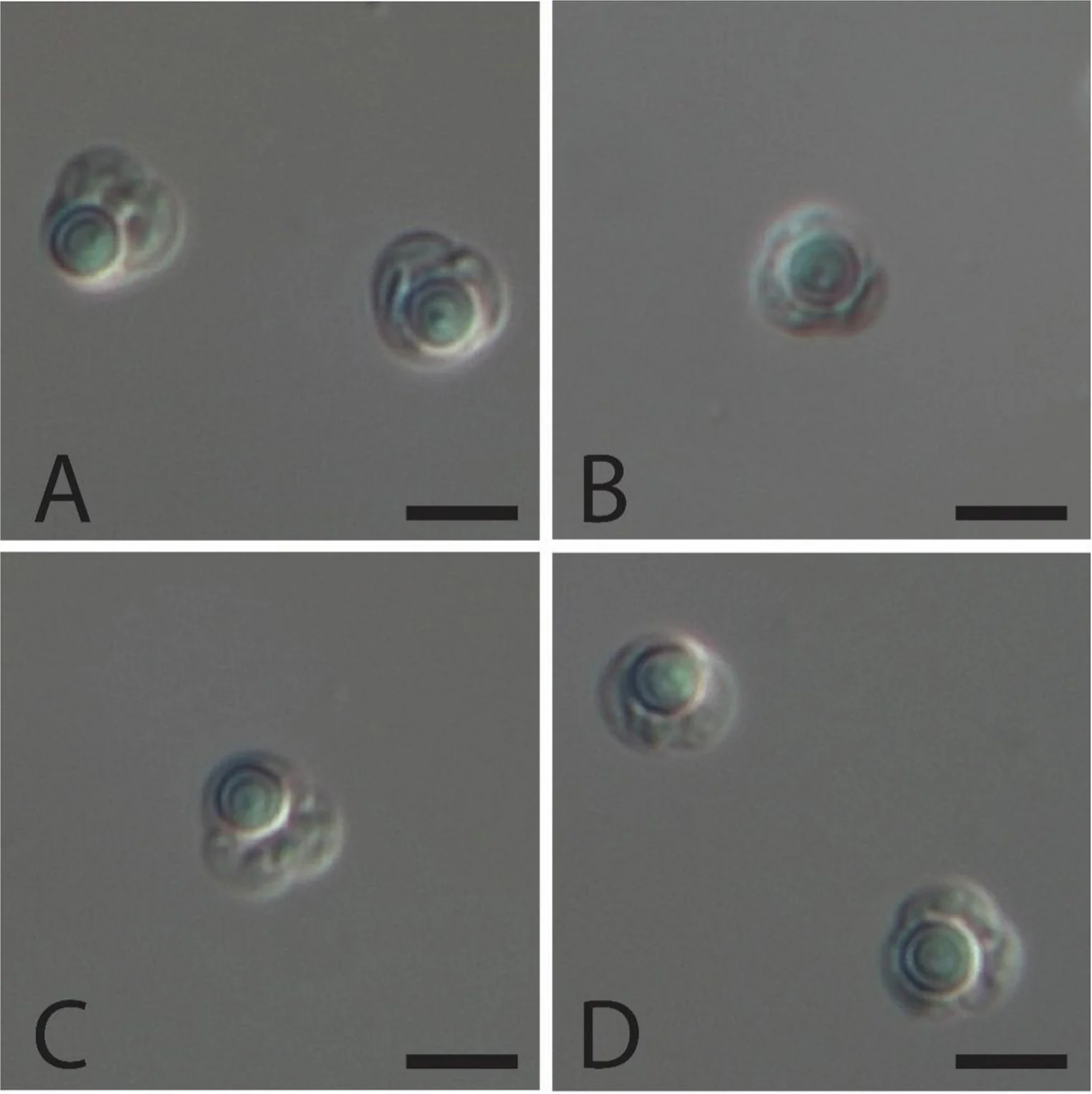
(**A**-**D**) Myxospores consistent in appearance with *Unicapsula seriolae* from yellowtail kingfish (*Seriolae lalandi*) tissue in wetmount under 1000x magnification. Nomarski. Bar = 5 μm

Myxospores consistent in appearance with *U. seriolae* were observed in wetmounts of a subset of tissues that were PCR positive for myxozoan DNA (Fig. 2). Specifically, the myxospores (*n* = 40) were subspherical and slightly lobed with a spore diameter of 6.05 μm (95% CI: 5.43–6.62 μm, 0.04 μm) (Table 1), composed of 3 shell valves, and possessed a large polar capsule with a diameter of 3.04 μm (2.37–3.35 μm, 0.04 μm) (Table 1). Myxospore dimensions were consistent with prior reports for *U. seriolae* (Lester 1982; Miller and Adlard 2013) (Table 1). For the mushy YTK from Broughton Island (Supplementary Table S3), no visible myxospores were observed in wet mounts, consistent with molecular results.

**Table 1:** Spore and polar capsule measurements (µm) of *Unicapsula seriolae* in yellowtail kingfish (*Seriola lalandi*) tissue

Source	Spore diameter (µm), (range, standard error), *n*	Polar capsule diameter (µm), (range, standard error), *n*
Present study	6.05 (5.43–6.62, 0.04), *n* = 40	3.04 (2.37–3.35, 0.04), *n* = 40
Lester (1982)	5.6 (4.9–6.3)	2.9 (2.1–3.5)
Miller and Adlard (2013)	6.1 (5.2–6.6)	3.4 (2.2–3.9)

### Molecular findings

#### Overall infection prevalence

**Fig. 3:**
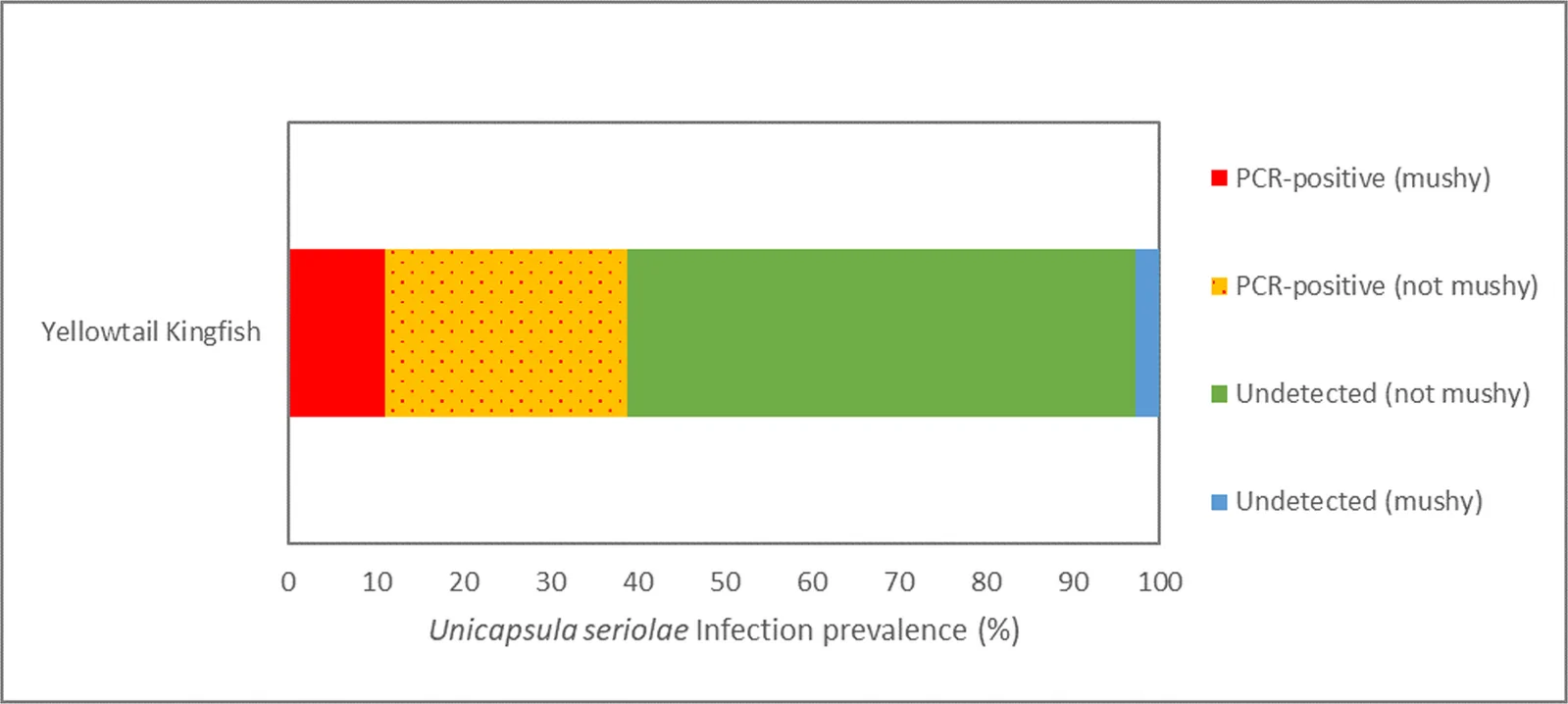
Unicapsula infection prevalence (%) in 36 wild yellowtail kingfish (Seriola lalandi) from New South Wales waters (2024–2025). Fish could be PCR-positive with mushy flesh on cooking (red), PCR-positive and not mushy on cooking (orange with red dots), PCR-undetected and not mushy on cooking (green), or PCR-undetected with mushy flesh as reported by the angler (blue)

Among the 36 wild YTK tested from NSW, 14 fish tested positive for myxosporean infection by PCR using the SSU and LSU primers (Supplementary Table S2). Subsequent DNA sequencing identified these positives as *U. seriolae* (38.9% infection prevalence, 95% CI: 23.1–56.5%) (Fig. 3). Only 28.6% of the 14 infected fish were reported as mushy by anglers (Fig. 3) and both PCR and morphology were unable to detect myxosporeans in one mushy YTK from Broughton Island, resulting in 80% of mushy fish (4/5) testing positive for *U. seriolae* (Supplementary Table S2).

Fisher’s exact tests indicated no strong univariable association between PCR detection and bioregion (*p* = 0.27), season (*p* = 0.085), or mushy flesh condition (*p* = 0.064) (Supplementary Table S2). Initial binomial GLM diagnostics indicated inflated standard errors and extreme coefficient estimates for the intercept and the mushy predictor, consistent with separation (Supplementary Table S4). Univariable Firth penalised logistic regression suggested a borderline association between mushy condition and infection status; non-mushy fish had lower odds of PCR-confirmed *U. seriolae* infection than mushy fish (OR = 0.16, 5% CI 0.01–1.03, *p* = 0.053) (Supplementary Table S5).

Of the 36 fish examined, 33 complete cases were retained for multivariable analysis following exclusion of samples with missing covariate data (Supplementary Table S3). PCR detection of *U. seriolae* was modelled using multivariable Firth penalised logistic regression as a function of bioregion, water temperature, fish length, and mushy flesh condition (Table 2). Full model estimates are provided in Supplementary Table S6.

The multivariable model provided weak overall evidence for an association between predictors and infection status (likelihood ratio test: χ² = 8.50, df = 4, *p* = 0.075; Wald test *p* = 0.216) (Table 2). Within the multivariable Firth‑penalised model fitted to 33 complete cases, mushy flesh was strongly associated with PCR-confirmed infection: non-mushy fish had substantially lower odds of infection compared to mushy fish (OR = 0.005, 95% CI: 0.000002–0.50, *p* = 0.021) (Table 2). In contrast, bioregion was not associated with infection status (*p* = 0.96) with similar odds (OR = 1.05, 95% CI: 0.17–7.39) of detection between the Manning Shelf and Hawkesbury Shelf (Table 2). Water temperature showed a negative but unsupported association with infection probability (*p* = 0.13) where each 1 °C increase in water temperature was associated with a 27% reduction in the odds of infection (OR = 0.73 per 1 °C increase, 95% CI: 0.41–1.09), and fish length was not associated with infection status after adjustment (*p* = 0.34) (OR = 0.96 per cm increase, 95% CI: 0.85–1.05) (Table 2).

**Table 2:** Multivariable Firth penalised logistic regression of Unicapsula seriolae PCR detection in yellowtail kingfish (Seriola lalandi) from New South Wales waters (2024–2025). Adjusted odds ratios (OR), 95% profile penalised likelihood confidence intervals (CI), and p-values from the multivariable model. The model was fitted to 33 complete cases following the exclusion of samples and removal of observations with missing covariate data (Supplementary Table S3). Overall model fit statistics were likelihood ratio test χ² = 8.50, *p* = 0.075; Wald test χ² = 5.79, *p* = 0.216

Predictor	Comparison / Unit	β (log-odds)	OR	95% CI (OR)	*p*-value
Bioregion	Manning vs. Hawkesbury	0.048	1.05	0.17–7.39	0.959
Water temperature	per 1 °C increase	-0.312	0.73	0.41–1.09	0.130
Length	per cm increase	-0.045	0.96	0.85–1.05	0.344
Mushy texture	Not mushy vs. mushy	-5.343	0.005	0.000002–0.50	0.021

### Spatio-temporal distribution of infection and phenotype

#### Spatial distribution

**Fig. 4:**
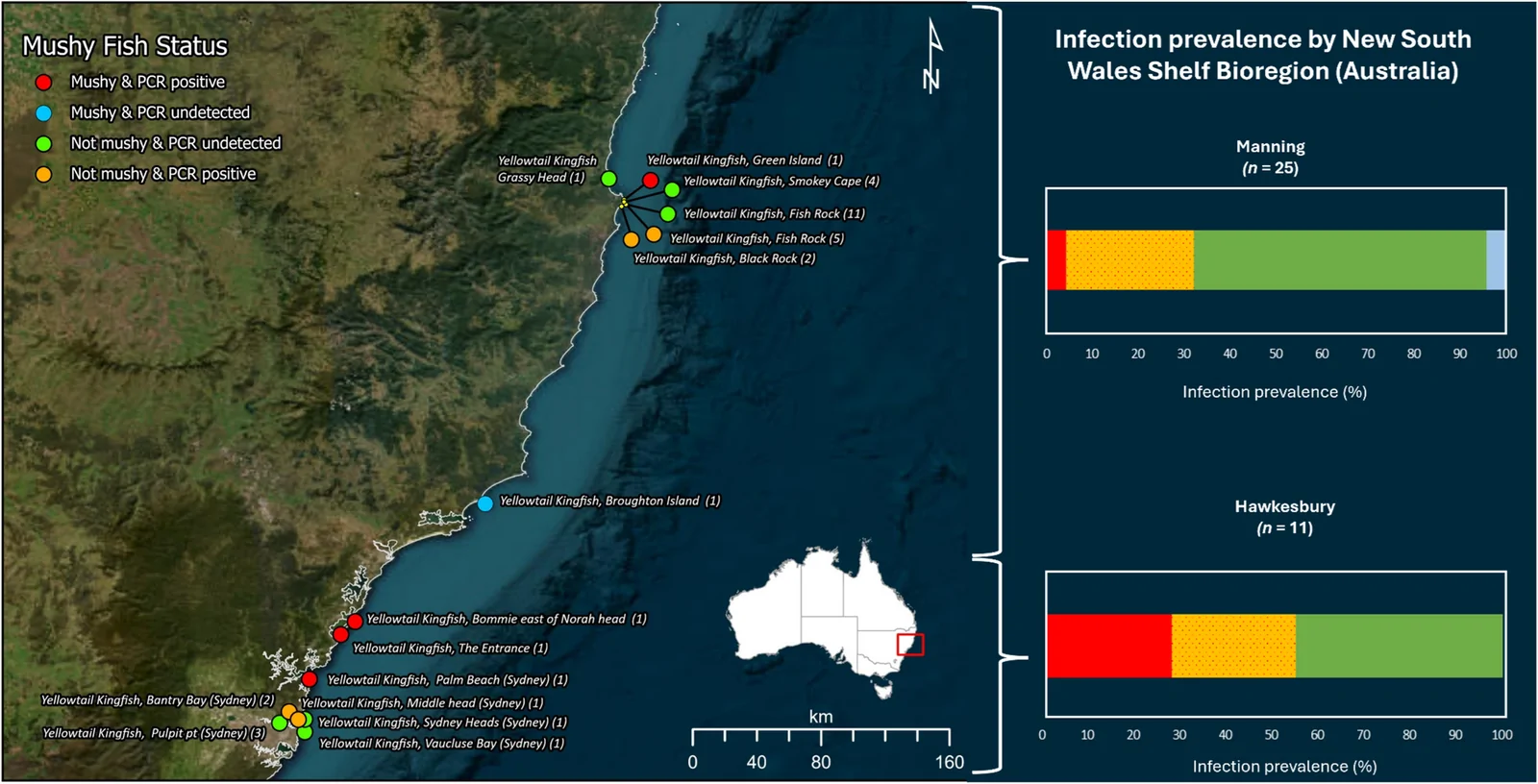
Spatial distribution of 36 yellowtail kingfish (*Seriola lalandi*) donated by recreational anglers landed in NSW, Australia (2024–2025). Fish could be PCR-positive for *Unicapsula seriolae* with either mushy flesh (red) or without mushy flesh (orange) as reported by anglers, or mushy fish could have no myxosporean parasites detected by PCR (blue), or fish that weren’t mushy could have no parasites detected by PCR (green). Twenty-five fish were landed in the northern Manning Shelf Bioregion (MSB) and had an infection prevalence of 32% for *U. seriolae* where 4% of PCR-positive fish were reported as having mushy flesh by anglers. Eleven fish were landed in the more southern Hawkesbury Shelf Bioregion (HSB) and had a higher infection prevalence of 54.5% for *U. seriolae*, and PCR-positive fish were equally distributed between mushy (27.3%) and non-mushy fish (27.3%). One mushy fish from Broughton Island had no myxosporean parasites detected by PCR or morphological analysis. Map produced with ArcGIS Pro 3.6.0

PCR detection of *U. seriolae* was observed in both bioregions sampled, indicating broad spatial occurrence across the study area (Fig. 4). Partitioning fish landed according to the NSW shelf bioregion resulted in a higher number of fish caught in the Manning Shelf Bioregion (MSB) compared to the Hawkesbury Shelf Bioregion (HSB) (69.4% (*n* = 25) and 30.5% (*n* = 11), respectively) (Fig. 4) (Supplementary Tabel S2). Fish landed in the southern HSB showed a higher infection prevalence with *U. seriolae* (54.5%) than fish landed further north in the MSB (32%) (Fig. 4) (Supplementary Tabel S2). In the HSB, PCR-positive fish were equally distributed between mushy and non-mushy individuals (27.3% each), whereas in the MSB, more PCR-positive fish (24.0%) did not exhibit the mushy phenotype, compared to 4.0% of mushy fish that were infected (Fig. 4) (Supplementary Tabel S2).

#### Temporal distribution

**Fig. 5:**
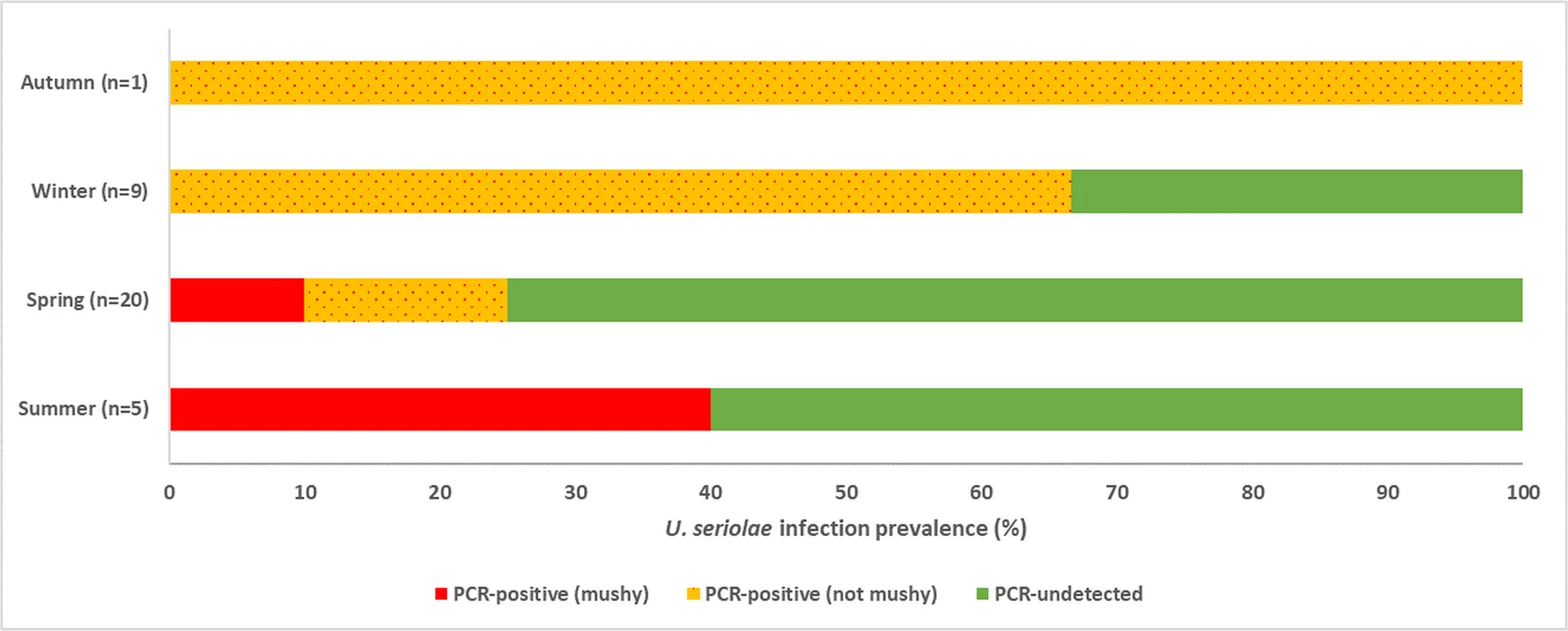
Infection prevalence of *Unicapsula seriolae* in 36 wild yellowtail kingfish (*Seriolae lalandi*) landed in NSW, Australia throughout Austral seasons, 2024*–*2025. Fish could be parasite-positive by PCR for *Unicapsula seriolae* with either mushy flesh (red) or parasite positive by PCR without mushy flesh (orange) as reported by anglers, or myxozoan parasites could remain undetected by PCR and fish were not mushy (green). For the Austral seasons of 2024, one fish was landed in autumn, nine fish were landed in winter, 20 fish were landed in spring and five were landed in the 2024*–*2025 summer. One YTK (Broughton Island) with reported mushy flesh was excluded from temporal analyses due to an unreported landing date; no myxosporeans were detected using morpho-molecular methods

Ongoing sampling throughout 2024–2025 revealed that *U. seriolae* was present throughout the calendar year (Fig. 5) (Supplementary Table S2 and S3), however, mushy flesh was only reported in fish during summer and spring (Fig. 5) and in larger fish (110–132 cm), while smaller infected fish (70 cm) remained asymptomatic (Supplementary Table S2 and S3). In summer, 40% of YTK tested positive for *U. seriolae* (*n =* 5), and all presented as mushy, while in spring, 25% of YTK were infected with *U. seriolae* (*n =* 20) which presented as both mushy (10%) and not mushy (15%) (Fig. 5). The single fish landed in autumn was positive for *U. seriolae* and did not have mushy flesh (Fig. 5). In winter (*n* = 9), 66.6% tested positive for *U. seriolae*, and did not have mushy flesh (Fig. 5). The date of landing for a single mushy, but PCR-undetected YTK from Broughton Island was not reported by the angler and season was unable to be accounted for.

### Comparison of *U. seriolae* infection prevalence with previous Australian studies

**Fig. 6:**
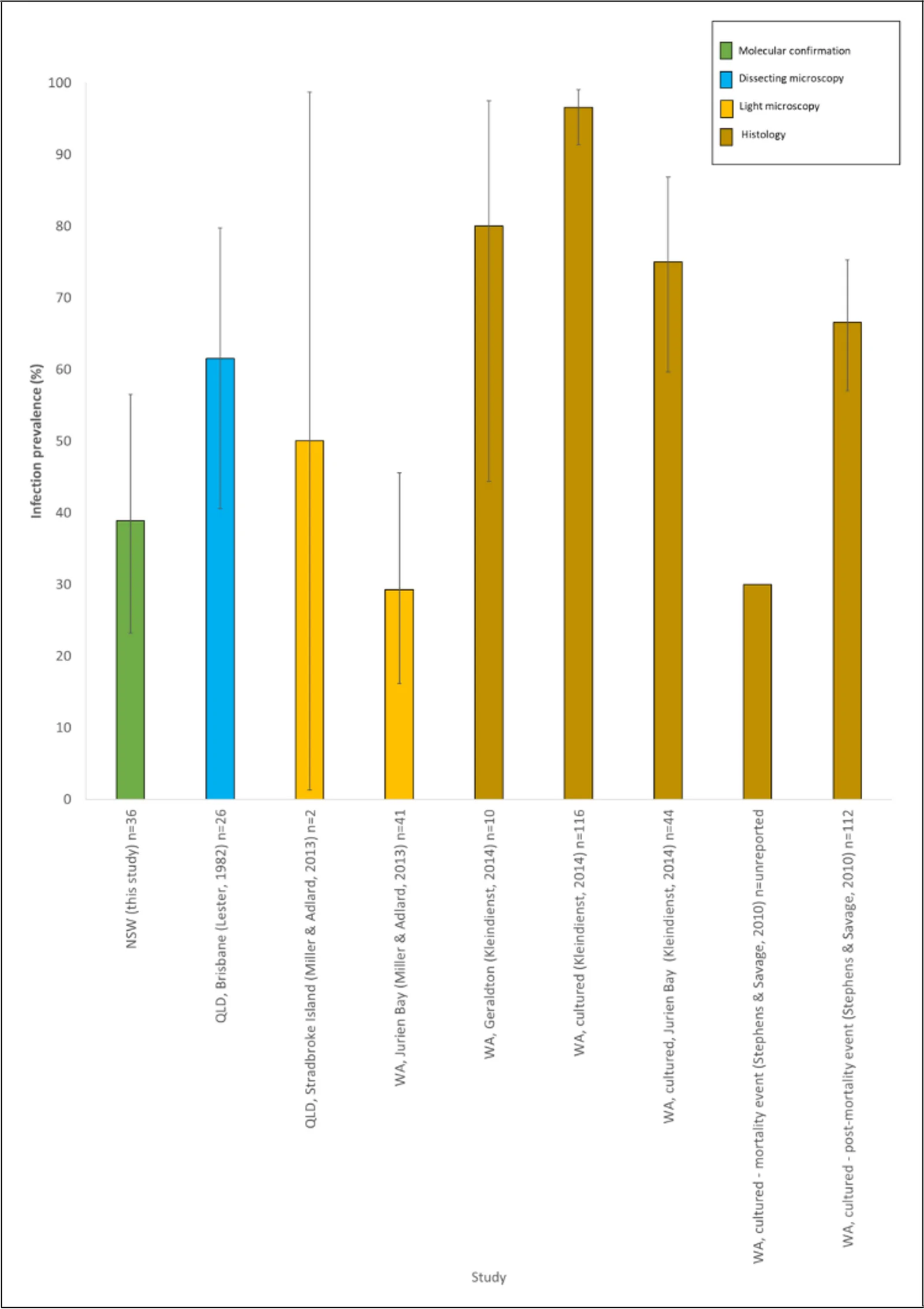
*Unicapsula seriolae* infection prevalence (95% confidence intervals (Clopper-Pearson) in yellowtail kingfish (*Seriolae lalandi*) landed across states in Australia – NSW (this study) 38.9% (95% CI: 23.1–56.5%) and for graphical comparison with previous Australian reports in QLD (Lester 1982; Miller and Adlard 2013), and WA (Kleindienst 2014; Miller and Adlard 2013; Stephens and Savage 2010). All fish were wild except for those denoted as cultured in WA. Historical records differ in diagnostic method as denoted by the legend and time as stated on x-axis; comparisons are descriptive only. Additional cultured fish (South Australia *n* = 2, Fremantle, WA *n* = 2) showed no evidence of infection on histological examination (Kleindienst 2014)

Wild YTK infection prevalence in previously published reports were 50% (CI: (1.3%–98.7%) *n* = 2) (Miller and Adlard 2013) and 61.5% (CI: 40.6%–79.8%, *n* = 26) (Lester 1982), in QLD, 80.0% (CI: 44.4%–97.5%, *n* = 10) in Geraldton, WA (Kleindienst 2014), 29.3% (CI: 16.1%–45.5%, *n* = 41) in Jurien Bay, WA (Miller and Adlard 2013) (Fig. 6). 96.6% (CI: 91.4%–99.1%, *n* = 116) and 66.6% (CI: 56.5%–74.7%, *n* = 112) of cultured fish from WA were infected (Kleindienst 2014; Stephens and Savage 2010) (Fig. 6).

### Citizen-science mapping of mushy flesh occurrence in yellowtail kingfish

A total of 48 georeferenced reports were submitted between October 2024 and November 2025 (Supplementary Table S7). YTK dominated submissions (85.4% of reports), followed by Mahi Mahi (*Coryphaena hippurus*) Linnaeus, 1758 (6.3%), Yellowfin Bream (*Acanthopagrus australis*) Günther, 1859 (4.2%), and single reports of Long Tom *(Strongylura leiurus)* Bleeker, 1851, and Mulloway (*Argyrosomus japonicus*) Temminck & Schlegel, 1844 (2.1% each) (Supplementary Table S7). Records clustered along the east coast from Noosa Heads (QLD) to Shellharbour (NSW); three Kingfish reports were outside QLD/NSW (Mornington Peninsula VIC and two WA sites). Of 39 fish with length and temperature data reported, most fish landed occurred at water temperatures of 21–30 °C (61.5%), and the 71–80 cm size class was most frequently reported (25.6%). Approximately 50% of reports described mushy flesh, with size-class analysis showing the highest prevalence in the 91–100 cm size class (80%) (Supplementary Table S7). Of the 44 reports that included the date fish were landed, seasonal reporting was spread across summer (31.82%), followed by spring (29.55%), winter (27.27%) and autumn (11.36%) (Supplementary Table S7). The distribution of mushy flesh across all reports (*n* = 44) according to season included 15.9% for both summer and winter, followed by spring at 13.6%, and 11.4% for autumn (Supplementary Table S7). When restricted to the fish reported as having mushy flesh (*n* = 25), the seasonal distribution was: autumn 5/25 (20%), winter 7/25 (28%), summer 7/25 (28%), and spring 6/25 (24%) (Supplementary Table S7). Expressed as a proportion of fish submitted in each season, the prevalence of mushy flesh in autumn was 5/5 (100%), winter 7/12 (58.3%), summer 7/14 (50%), and spring 6/13 (46.1%) (Supplementary Table S7).

#### Angler and consumer observations of mushy flesh occurrence and associated impacts

Of the 48 records submitted, 27 records included comments (Supplementary Table S7) which revealed several dominant themes that reinforce the mapped patterns (Table 3): reports of mushy flesh on cooking (65%), attribution to seasonality or a temperature signal (35%), shallower nearshore locations (27%), inconsistent handling/storage concerns and perceptions, and impacts to consumers (23%), and stakeholder concerns and engagement including direct offers to provide samples or freezer access (31%). Several comments (Table 3, Supplementary Table S7) described within-trip heterogeneity (one fish with mushy flesh, another fish without mushy flesh) and a small but consistent stream of anglers reported recent increases in local occurrences. Anglers’ comments span a variety of impacts and document the mushy flesh issue in YTK in Western Port Bay, VIC, in the 1950s (Table 3, Supplementary Table S7), causing them to cease fishing for the species. Furthermore, a report also documented market-based impacts in the 1980’s in NSW, where the consumer may have received a refund (Table 3, Supplementary Table 7).

**Table 3:** Of the 48 records submitted to the Mushy Fish StoryMap, 27 records included comments which revealed several dominant themes that reinforce the mapped patterns (Supplementary Table S7): reports of mushy flesh on cooking (65%), attribution to seasonality or a temperature signal (35%), shallower nearshore locations (27%), inconsistent handling/storage concerns and perceptions, and impacts to consumers (23%), and stakeholder engagement and concerns (31%)

Theme	Observations	Anonymised angler comment 1	Anonymised angler comment 2
Seasonality and temperature signal	Multiple comments explicitly note higher occurrences in Austral spring–summer and attribute this to warmer water.	*“Half of the kingfish caught in end Dec to mid Jan have been mushy when cooked.”*	*“Kingfish go mushy ’cause of the warmer water.”*
Nearshore/depth association	Fishers repeatedly describe shallower coastal grounds as the sites where mushy fish are encountered.	*“These kingfish were caught in the 1950s in Western Port Bay Victoria; they were inedible as they turned to mush. They were so bad*,* we stopped fishing for them. Kingfish caught off Lord Howe and Montague Islands are good.”*	*“Have caught several kingfish and bream around Coffs Harbour with similar mushy flesh.”*
Patchy, fine-scale infection	Several reports describe mixed outcomes from the same trip (one fish mushy, another firm).	*“We caught 3 kingfish that day in roughly the same area. Only 1 was mushy. We observe that roughly 1 in 3 kingfish in this area are mushy. Anecdotal evidence.”*	*“Five years ago*,* you would be lucky to get an infected fish. These days it’s 1 in 4 at the least*,* I would say!”*
Handling and food safety perceptions	Comments vary on storage/handling as a cause (some note immediate icing/freezing without prevention), and many note that sashimi/raw preparations appear unaffected while cooked flesh becomes mushy.	*“Absolutely fine as sashimi or ceviche; once under heat*,* it became very mushy.”*	*“Fish filleted as per normal and felt fine. Turned to mush when cooking. Bitter taste.”*
Flow-on impacts to markets and consumers	Other comments note that the mushy flesh issue reached the consumer through the markets.	*“I remember buying one from [a NSW-based] market in the early 1980s which was the same. We mentioned it to the vendor. I think we got our money back.”*	*“We were at [a] restaurant. We had one*,* sent back to kitchen. They came back 10 min later and said the same thing happened with a second kingfish. We had no idea this problem existed.”*
Stakeholder engagement	Anglers frequently offer samples or contact details.	*“I had the fillets in the fridge for 3 days before I froze them*,* would they be of use for analysis? Call on.”*	*“Keen to help your research with 3 of my stories; ring if you wish.”*
Stakeholder concern	Anglers express frustration and disgust, report behavioural changes (e.g., avoiding fishing certain areas).	*“We will no longer fish for them as it is very off putting. Lake Macquarie NSW.”*	*“Finding kudoa more prominent in our area… very disappointing to dispatch these fish unnecessarily.”*

## Discussion

### Morpho-molecular confirmation and prevalence in NSW

This study provides the first morpho-molecular confirmation of *Unicapsula seriolae* in YTK from NSW, at a prevalence of 38.9%. Spore morphology, including spore shape, polar capsule dimensions and polar filament arrangement, was consistent with the original description by Lester (1982) and was further supported by molecular identification, providing robust confirmation of the species identity. The prevalence observed in this study demonstrates that *U. seriolae* is present in YTK from the Manning and the Hawkesbury shelf bioregions in NSW and provides a valuable baseline for future surveillance and epidemiological investigations.

Previous studies in NSW either reported no evidence of *U. seriolae* based on histological examination (Hutson et al. 2007a, b) or described the cooked-mushiness condition based on gross observations without parasite confirmation (Forrest 2019; Smith et al. 1991). This raises the possibility that *U. seriolae* may have been historically present in NSW, but remained undetected at low prevalence or intensity in earlier studies (Forrest 2019; Hutson et al. 2007a, b; Smith et al. 1991),The present findings suggest that differences in diagnostic methodology, may have contributed to differing observations among studies. More broadly, these results highlight the importance of combining complementary diagnostic approaches to improve the detection and accurate identification of myxozoan parasites, particularly where infections may be focal, of low intensity, or difficult to detect using a single approach.

### The parasite-phenotype mismatch: decoupling infection from mushy flesh

The mushy flesh phenotype was an unreliable indicator of infection: it occurred in only 28.6% of the 14 infected YTK, while 71.4% of infected fish remained asymptomatic on cooking. These PCR results were corroborated by wet-mount microscopy and sequencing, indicating that PCR-positive, phenotypically normal fish reflect genuine subclinical infections rather than false positives. Infection therefore appears necessary but not sufficient for the mushy flesh phenotype, which instead presents as a conditional outcome and is consistent with observations in other myxozoan-affected Australian pelagic fisheries (Bolin et al. 2021).

This variability was illustrated within a single catch where one fish developed mushy flesh out of three YTK landed together from the same school under identical conditions. The manifestation of infection-induced phenotypes depends on infection intensity, co-infections, parasite developmental stage, host condition, post-harvest handling and environmental context, which likely all interact to determine expression (Nadler et al. 2023).

Although these patterns are consistent with a parasite-mediated mechanism in most (4/5) mushy fish here, Koch’s postulates (Hutson et al. 2023; Koch 1884) remain unfulfilled, and causality has not been experimentally confirmed. Notably, one mushy YTK from Broughton Island showed no evidence of myxosporean infection by either PCR or microscopy, indicating that mushy flesh can arise from causes other than *U. seriolae*, potentially including storage/handling conditions or other microorganisms, which were not assessed here. Alternatively, the methods applied here may have missed infections that were focal or of low intensity. Reliance on the mushy flesh phenotype alone therefore substantially underestimates true *U. seriolae* infection prevalence in YTK.

### Spatio-temporal dynamics of infection and phenotypic expression

There was no statistical association between infection prevalence and season or bioregion (Fisher’s exact test; multivariable Firth model), although seasonal inference is limited by uneven sampling (autumn, *n* = 1) and one fish with an unreported landing date. At 38.9%, prevalence in NSW falls within the range reported for wild YTK elsewhere in Australia of 29.3%-80.0% (Kleindienst 2014; Lester 1982; Miller and Adlard 2013), and is lower than QLD (61.5%; Lester 1982) and Geraldton, WA (80%; Kleindienst 2014), but higher than Jurien Bay, WA (29.3%; Miller and Adlard 2013). These differences likely reflect a mix of host demographics, regional thermal regimes, methodological heterogeneity and spatio-temporal variability across studies using microscopy, histology, or molecular testing, rather than genuine differences in parasite occurrence, and do not support strong regional conclusions (Kleindienst 2014; Lester 1982; Miller and Adlard 2013).

The absence of clear spatial or seasonal structuring may reflect the high genetic connectivity and extensive pelagic mobility of YTK along the east coast (Clarke et al. 2024; Goddard et al. 2024, 2026; Miller et al. 2011), which could facilitate widespread transmission (Hallett et al. 2015; Manlove et al. 2022; Tadiri et al. 2018), and/or fine-scale spatial heterogeneity in transmission not captured at the bioregional scale (Bolin et al. 2024; Giulietti et al. 2022a). However, this pattern could equally reflect limited statistical power from a small sample size (*n* = 36), rather than genuine ubiquity, and should be treated as a hypothesis for larger-scale surveillance rather than a confirmed feature of the parasite’s distribution. 

The parasite may occur more broadly across south-eastern Australia than currently documented, supported by the confirmed long-distance movements of the eastern YTK stock between SA and NSW (up to 1,800km Clarke et al. 2024). This widespread distribution is further influenced by poleward range shifts driven by changing oceanographic conditions and host movement patterns (Champion et al. 2019; Giulietti et al. 2022a; Goddard et al. 2026; Phillips et al. 2022; Shamsi and Barton 2024), notwithstanding earlier reports of its apparent absence in SA based on limited histological sampling in aquaculture (*n* = 2) (Diggles 2011; Kleindienst 2014).

Beyond host-mobility, transmission dynamics can also depend on other ecological factors (e.g. biotic and abiotic factors such as intermediate hosts and environmental conditions) (Alama-Bermejo et al. 2013; Daversa et al. 2017; Díaz-Morales et al. 2026; Lauringson et al. 2023; Ray and Bartholomew 2013; Schmidt-Posthaus and Wahli 2015). Consistent with temperature- and depth-related transmission patterns, fish from shallower, warmer sites are reported to have higher infection rates both in Australia and globally (Bolin et al. 2024; Giulietti et al. 2022a). Episodic warm, shallow hotspots (driven by the east Australian current (EAC)) and transient anomalies) may also elevate local transmission risk (Bolin et al. 2024), which may reflect the higher point prevalences observed in QLD (61.5%) (Lester 1982) compared to NSW (38.9%).

The parasite *U. seriolae* was detected across the full sampling year, spanning a sea surface temperature (SST) range of 16.0–26.1 °C, whereas the mushy flesh phenotype was confined to spring and summer (~ 20–26 °C). Notably, infected fish at comparable warm SSTs in autumn (23–23.7 °C) and infected fish in winter (16–19.6 °C) remained asymptomatic, indicating that elevated SST and location at capture alone are insufficient to explain expression — a pattern also seen in other Australian pelagic fisheries affected by temperature-associated soft-flesh conditions (Bolin et al. 2024). SST values were partly supplemented from public data where angler-reported readings were unavailable, introducing some uncertainty around the precise thresholds identified. Comparable temporal fluctuation and intermittent absence have been documented for other NSW marine fish parasites, such as anisakid nematodes, reflecting complex interactions between environmental conditions, host distribution, and other ecological drivers (Shamsi 2021; Shamsi et al. 2018). The current widespread distribution of *U. seriolae* across the two shelf bioregions in NSW therefore likely reflects a combination of improved detection and dynamic oceanographic processes, rather than a single explanatory mechanism.

Phenotypic expression also differed markedly between bioregions (4% of Manning Shelf fish vs. 27.3% of Hawkesbury Shelf fish were mushy), suggesting that local conditions in the southern shelf system may have been more conducive to activating the parasite’s proteolytic potential during this sampling window, potentially via differences in parasite intensity, co-infections, host condition, acute environmental stressors, or post-harvest handling, or through regional differences in fish age/size structure (Nadler et al. 2023; Stewart et al. 2026). Given the small samples of fish landed in the Hawkesbury (*n* = 11) and the Manning (*n* = 25), this divergence should be treated as preliminary. Infection status likely reflects cumulative lifetime exposure across habitats, whereas phenotypic expression may instead reflect recent, local conditions (Nadler et al. 2023), where infection (which is largely constant) is separate from the risk of expression (apparently conditional).

### Citizen-science insights into mushy flesh occurrence and impacts

Citizen-science reporting provided complementary spatial and temporal context but cannot substitute for molecular diagnosis as phenotype alone cannot estimate infection prevalence. Angler-reported mushy fish were generally caught at warmer temperatures (21–30 °C), broadly consistent with the spring–summer restriction of the phenotype in the molecular dataset (20–26 °C), but reports also included mushy fish in autumn (100%, *n* = 5, from shallow coastal sites such as Terrigal and Black Rocks, NSW, and Shelley Beach, WA) and winter (58%) — proportionally higher than summer (50%) or spring (46%). This apparent inconsistency with the molecular data reinforces that temperature and infection alone do not fully account for phenotypic expression and likely reflects the diverse recent life histories of highly mobile, connected YTK along the east coast (Clarke et al. 2024; Goddard et al. 2024, 2026; Nadler et al. 2023).

Retrospective angler accounts report encounters with mushy YTK as far back as the 1950s (Western Port Bay, VIC) and the 1980s (NSW markets), suggesting intermittent occurrence for decades. However, as these accounts do not confirm *U. seriolae* presence, and as most infections in this study were asymptomatic, many historical reports of mushy flesh may reflect a broader, multi-factorial etiology rather than being exclusively attributable to this parasite.

These reports also provide the first documentation of the condition’s practical impacts on NSW anglers and consumers: inedible catches, wasted effort, diminished enjoyment, eroded consumer confidence, and unnecessary fish wastage, with some anglers reporting they had stopped fishing particular areas or species altogether. Given that NSW supports Australia's largest recreational (~ 114 t landed in 2019/20) and commercial catch (65 t in 2021/22) for the species (Hughes et al. 2023; Hughes 2023), and that recreational fishing more broadly generates an estimated ~$3.4 billion and ~ 14,000 FTE jobs for the state (State of New South Wales, 2026), sustained discouragement among anglers could potentially have downstream effects on coastal tourism and businesses reliant on the fishery’s reputation, although this remains an inference from qualitative reports rather than a measured outcome. Integrating stakeholder reporting with molecular surveillance offers a scalable approach for flagging periods or locations of elevated risk to inform adaptive harvest and handling advice.

### Implications for fisheries, seafood safety and aquaculture

The lack of market-based diagnostics is a critical gap for seafood safety and quality assurance, as infected and uninfected YTK are organoleptically indistinguishable until cooked, and 71.4% of infections in this study were asymptomatic, phenotypic screening alone will let infected fish enter the supply chain undetected, as a raw ready-to-eat product. While *U. seriolae* is not considered a major zoonotic parasite and its implications for human health remain unclear, food-borne illness associated with *U. seriolae* in raw greater amberjack (*Seriola dumerili*) has been documented in Japan (Kodo et al. 2026; Ohnishi et al. 2016, 2018) and *Kudoa septempunctata* has caused confirmed food-poisoning outbreaks via aquacultured olive flounder (Kawai et al. 2012). Spore burden was not systematically quantified in this study, but these precedents suggest *U. seriolae* infection in YTK warrants consideration from a food-safety and quality-assurance perspective for both domestic and export raw markets (Rigos et al. 2021; Tomochi et al. 2014).

Southeastern Australian waters are warming around three times the global average due to an intensifying EAC (Frusher et al. 2013; Malan et al. 2021; Phillips et al. 2022), and marine heatwaves are projected to grow in frequency and intensity, posing a broader risk to Australia’s ~$3.45 billion commercial seafood sector (Brodie et al. 2026). If, as we hypothesise, host thermal stress contributes to phenotypic expression, warming could increase both host susceptibility to disease more generally (Burge et al. 2014; Garren et al. 2014; Harvell et al. 2002; Holzer et al. 2021; Tout et al. 2015) and the frequency of mushy flesh specifically, although this remains to be tested. The population-level consequences of infection (effects on fish health, condition, and recruitment) are unresolved; myxosporeans elicit host immune responses (Gómez et al. 2014) and include established fish pathogens such as *Kudoa yasunagai* in YTK (Banu and Rathinam 2023; Holzer et al. 2021; Ishimaru et al. 2014; Shin and Shirakashi 2017), and *U. seriolae* has previously been implicated, alongside other co-occurring pathogens, in mass mortality events in WA sea-cage YTK (Stephens and Savage 2010). Clarifying the drivers of both infection and expression will help distinguish the risk of infection (likely constant) from the risk of expression (apparently conditional) and support the revision of adaptive harvest and management strategies (Duggan 2025; LTHSWG 2024) as parasite distribution potentially shifts under climate change (Shamsi and Barton 2024).

These findings also carry implications for YTK aquaculture. No large-scale operation currently exists on Australia’s east coast, but past pilot trials in NSW (Booth et al. 2010) and WA (Kleindienst 2014; Stephens and Savage 2010; Stephens 2016) and an ongoing Bass Strait trial mean that wild YTK populations, as a likely reservoir of *U. seriolae*, may potentially pose a biosecurity risk. WA aquaculture data show substantially higher infection prevalence (66–96%; Kleindienst 2014; Stephens and Savage 2010) than reported here, plausibly reflecting the higher host densities and reduced ability of caged fish to avoid localised thermal stress, both of which may favour transmission and compromise the host’s capacity to suppress virulence (Hutson 2007; Hutson et al. 2007a, b; Kleindienst 2014; Stephens and Savage 2010). Robust biosecurity and site-selection protocols that account for optimal thermal windows (Clarke et al. 2024), will be important for managing this risk as warm-water anomalies such as those projected for south-eastern Australia warming hotspots (Frusher et al. 2013; Malan et al. 2021; Phillips et al. 2022) may extend to sea-cage systems.

### Limitations and caveats

These findings should be interpreted with caution given several methodological constraints. Sampling was opportunistic and uneven, relying on angler donations and citizen-science reports, resulting in limited seasonal representation and missing data for some cases (e.g., the Broughton Island YTK). Supplementing missing in situ SST measurements with public data introduces further uncertainty around the temperature relationships reported. The association between mushy texture and PCR-confirmed infection should also be interpreted cautiously as it is based on a small number of infected fish (*n* = 14) and 33 complete cases, and the extreme odds ratios obtained suggest near-separation, which can inflate effect estimates even after Firth penalisation. Potential confounding by host length, which correlated with mushy status, and reporting bias in citizen-science submissions, where unusual events are more likely to be logged than normal catches may also affect the prevalence and effect-size estimates presented.

### Future directions

We propose a hypothetical mechanism, not tested here, to explain the potential drivers underpinning the manifestation of mushy flesh. As YTK possess a broad thermal range (11–27 °C) with an apparent affinity for ~ 17–21 °C (Goddard et al. 2026; Pirozzi and Booth 2009), individuals operating beyond an optimal thermal threshold (~ 22 °C; Clarke et al. 2024; Pirozzi and Booth 2009) for a sustained period may accrue a bioenergetic cost that, combined with parasite load, co-infections, and host condition, could push a latent infection toward the heat-activated, protease-mediated tissue breakdown associated with other myxozoans (Alama-Bermejo et al. 2022; Miller 2024; Samaranayaka et al. 2007; Tsuyuki 1982) once the fish is cooked. Under this model, expression would depend on an individual’s recent cumulative thermal exposure (Goddard et al. 2026; Nadler et al. 2023) rather than the instantaneous SST at capture. Consistent with this model, infected YTK landed in summer and spring (20–26 °C), were the only fish in this study that developed mushy flesh, while asymptomatic infected fish were recorded at comparably warm autumn SSTs, when highly mobile YTK may have only recently moved into a warm pocket from cooler water as the EAC drives warm shelf incursions southward (Brodie et al. 2026; Champion et al. 2019; Goddard et al. 2026; Oliver and Holbrook 2018; Sobral et al. 2024). Heritable variation in thermal tolerance documented elsewhere in YTK (Munday et al. 2019) could plausibly contribute to individual differences in this threshold, but this remains speculative and was not tested here. Instead, we highlight it as a direction for future physiological and molecular work rather than an established mechanism.

## Conclusion

In conclusion, this study provides the first morpho-molecular confirmation that *Unicapsula seriolae* is widespread in YTK from the Manning and the Hawkesbury shelf bioregions, at an infection prevalence of 38.9%. Together with historical records from other regions, our findings suggest that the parasite is likely to occur across both genetic populations of YTK in Australia. The discovery of a parasite-phenotype mismatch, where 71.4% of infected fish remained asymptomatic, demonstrates that visual assessment of mushy flesh substantially underestimates the true extent of infection, and that its expression is a conditional outcome shaped by interacting host, parasite and environmental factors rather than a direct consequence of infection. Given the parasite’s association with mass-mortality events in aquaculture and with food-borne illness elsewhere, *U. seriolae*’s potential role as an opportunistic pathogen in YTK warrants further investigation, particularly under a changing climate.

## Electronic Supplementary Material

Below is the link to the electronic supplementary material.

Supplementary Material (DOCX 113 KB)

## Data Availability

Data is available in the supplementary file and will be made available upon request to the corresponding author.

## References

[CR1] Alama-Bermejo G, Šíma R, Raga JA, Holzer AS (2013) Understanding myxozoan infection dynamics in the sea: seasonality and transmission of Ceratomyxa puntazzi. Int J Parasitol 43(9):771–780 10.1016/j.ijpara.2013.05.003 23747926

[CR2] Alama-Bermejo G, Bartošová-Sojková P, Atkinson SD, Holzer AS, Bartholomew JL (2022) Proteases as therapeutic targets against the parasitic Cnidarian Ceratonova shasta: characterization of molecules key to parasite virulence in salmonid hosts. Front Cell Infect Microbiol 11. 10.3389/fcimb.2021.804864 PMC8777295 35071050

[CR3] Banu H, Rathinam RB (2023) Myxozoan fish diseases: possible treatment and zoonoses. J Parasit Dis 47(2):215–223. 10.1007/s12639-023-01568-9 PMC10182238 37193499

[CR4] Bartholomew JL, Kerans B (2015) Risk Assessments and Approaches for Evaluating Myxozoan Disease Impacts. In: Okamura B, Gruhl A, Bartholomew JL (eds) Myxozoan evolution, ecology and development (pp. 379–395). Springer International Publishing. 10.1007/978-3-319-14753-6_20

[CR5] Bolin JA, Cummins SF, Mitu SA, Schoeman DS, Evans KJ, Scales KL (2021) First report of Kudoa thunni and Kudoa musculoliquefaciens affecting the quality of commercially harvested yellowfin tuna and broadbill swordfish in Eastern Australia. Parasitol Res 120(7):2493–2503. 10.1007/s00436-021-07206-8 34115215

[CR6] Bolin JA, Evans K, Schoeman DS, Spillman CM, Moore TS, Hartog JR, Cummins SF, Scales KL (2024) A warming western boundary current increases the prevalence of commercially disruptive parasites in broadbill swordfish. Fish Oceanogr 33(4). 10.1111/fog.12669

[CR7] Booth MA, Allan GL, Pirozzi I (2010) Estimation of digestible protein and energy requirements of yellowtail kingfish Seriola lalandi using a factorial approach. Aquaculture 307(3):247–259. 10.1016/j.aquaculture.2010.07.019

[CR8] Born-Torrijos A, Kosakyan A, Patra S, Pimentel-Santos J, Panicucci B, Chan JTH, Korytar T, Holzer AS (2022) Method for isolation of myxozoan proliferative stages from fish at high yield and purity: an essential prerequisite for in vitro, in vivo and genomics-based research developments. Cells 11(3). 10.3390/cells11030377 PMC8833907 35159187

[CR9] Bray DJ (2025) *Seriola lalandi*. Museums Victoria, OzFishNet Community. Retrieved 22 Nov from https://fishesofaustralia.net.au/home/species/1662

[CR10] Brodie S, Blamey LK, Bugnot A, Bustamante RH, Blanchard JL, Buhagiar L, Fulton EA, Hobday AJ, Plagányi ÉE, Richardson AJ, Matear R, Woolley SNC, Darbyshire R (2026) Climate vulnerability of Australian seafood systems: a national assessment of exposure and adaptive capacity. Fish Oceanogr n/a(n/a). 10.1111/fog.70058

[CR11] Burge CA, Eakin M, Friedman C, Froelich CS, Hershberger B, Hofmann PK, Petes EE, Prager LE, Weil KC, E., Willis BL (2014) Climate change influences on marine infectious diseases: implications for management and society. Annual Rev Mar Sci 6(1):249–277 10.1146/annurev-marine-010213-135029 23808894

[CR12] Chai X, Li X, Lu R, Clarke S (2009) Karyotype analysis of the yellowtail kingfish (Seriola lalandi)(Perciformes: Carangidae) from South Australia. Aquac Res 40(15):1735–1741. 10.1111/j.1365-2109.2009.02278.x

[CR13] Champion C, Hobday AJ, Zhang X, Pecl GT, Tracey SR (2019) Changing windows of opportunity: past and future climate-driven shifts in temporal persistence of kingfish (Seriola lalandi) oceanographic habitat within south-eastern Australian bioregions. Mar Freshw Res 70(1):33. 10.1071/mf17387

[CR14] Clarke TM, Whitmarsh SK, Jaine FRA, Taylor MD, Brodie S, Payne NL, Butcher PA, Broadhurst MK, Davey J, Huveneers C (2024) Environmental drivers of yellowtail kingfish, (*Seriola lalandi*) activity inferred through a continental acoustic tracking network. Aquat Conservation: Mar Freshw Ecosyst 34(1). 10.1002/aqc.4019

[CR15] Daversa D, Fenton A, Dell A, Garner T, Manica A (2017) Infections on the move: how transient phases of host movement influence disease spread. Proc Royal Soc B: Biol Sci 284(1869):20171807 10.1098/rspb.2017.1807 PMC5745403 29263283

[CR16] Díaz-Morales DM, Atkinson SD, Boyd D, Commisso G, Christner SR, Jones I, Leslie KL, Thirtyacre J, Whalen CJ, Kuris A (2026) Stressor interactions affect myxozoan abundance in a 42-year dataset from the Pearl River, Louisiana, USA. Parasitology 153(5):654–666 10.1017/S0031182026101917 PMC13315210 41877365

[CR17] Diggles B (2011) Risk analysis – aquatic animal diseases associated with domestic bait translocation. 297 FRDC project no. 2009/072. https://share.google/ErBJaHoJmOEyKseiz

[CR18] Duggan J (2025) 7/09/2025). Opinion: NSW falls behind on kingfish management. Fish World. https://fishingworld.com.au/news/opinion-nsw-falls-behind-on-kingfish-management/

[CR19] Forrest AJ (2019) Identification of muscle parasite in Yellowtail Kingfish (Seriola lalandi) and Mahi Mahi (Coryphaena hippurus), and determination as to the efficacy of noninvasive screening technology for the purpose of identifying infected fish in a commercial fish processing environment. Progress Report preceding termination of the project. FRDC. https://www.frdc.com.au/sites/default/files/products/2017-020-DLD.pdf

[CR20] FRDC. (2023), June 20 *Open call for R&D investment opportunities* FRDC. https://www.frdc.com.au/call-funding-opportunities/june-2023-open-call-rd-investment-opportunities#toc-development-of-in-situ-detection-methods-kudoa-parasite-for-kingfish-and-mahi-mahi

[CR21] Froese R, Pauly D (2025) FishBase World Wide Web electronic publication. https://www.fishbase.org

[CR22] Frusher SD, Hobday AJ, Jennings SM, Creighton C, D’Silva D, Haward M, Holbrook NJ, Nursey-Bray M, Pecl GT, van Putten EI (2013) The short history of research in a marine climate change hotspot: from anecdote to adaptation in south-east Australia. Rev Fish Biol Fish. 10.1007/s11160-013-9325-7

[CR23] Garren M, Son K, Raina J-B, Rusconi R, Menolascina F, Shapiro OH, Tout J, Bourne DG, Seymour JR, Stocker R (2014) A bacterial pathogen uses dimethylsulfoniopropionate as a cue to target heat-stressed corals. ISME J 8(5):999–1007. 10.1038/ismej.2013.210 PMC3996689 24335830

[CR24] Giulietti L, Karlsbakk E, Cipriani P, Bao M, Storesund JE, Marathe NP, Levsen A (2022a) Long-term investigation of the ‘soft flesh’ condition in Northeast Atlantic mackerel induced by the myxosporean parasite Kudoa thyrsites (Cnidaria, Myxozoa): Temporal trends and new molecular epidemiological observations. Fish Res 248. 10.1016/j.fishres.2021.106221

[CR25] Goddard BK, Guillemin TA, Schilling HT, Hughes JM, Brodie S, Green CP, Harcourt R, Huveneers C, Ierodiaconou D, Suthers IM, Taylor MD, Tracey SR, Camilieri-Asch V, Clarke TM, Dwyer RG, Hilbert C, Holdsworth J, Mitchell J, Pepperell J, Jaine FRA (2024) Half a century of citizen science tag-recapture data reveals stock delineation and cross-jurisdictional connectivity of an iconic pelagic fish. Rev Fish Biol Fish 34(4):1433–1449. 10.1007/s11160-024-09880-0

[CR26] Goddard B, Schilling HT, Hughes JM, Taylor MD, Ierodiaconou D, Harcourt R, Brodie S, Huveneers C, Green CP, Tracey SR, Suthers IM, Guillemin TA, Jaine FRA (2026) Extensive pelagic habitat use by adult yellowtail kingfish (Seriola lalandi) in the Tasman Sea. Can J Fish Aquat Sci. 10.1139/cjfas-2025-0343

[CR27] Gómez D, Bartholomew J, Sunyer JO (2014) Biology and mucosal immunity to myxozoans. Dev Comp Immunol 43(2):243–256. 10.1016/j.dci.2013.08.014 PMC4216934 23994774

[CR28] Hallett SL, Hartigan A, Atkinson SD (2015) Myxozoans on the move: dispersal modes, exotic species and emerging diseases. Myxozoan evolution, ecology and development. Springer, pp 343–362

[CR29] Harvell CD, Mitchell CE, Ward JR, Altizer S, Dobson AP, Ostfeld RS, Samuel MD (2002) Climate warming and disease risks for terrestrial and marine biota. Science 296(5576):2158–2162 10.1126/science.1063699 12077394

[CR30] Heinze G, Ploner M, Dunkler D, Southworth H, Heinze MG (2025) Package ‘logistf’. Statist Med 21:2409–2419. 10.1002/sim.1047

[CR31] Holzer AS, Piazzon MC, Barrett D, Bartholomew JL, Sitja-Bobadilla A (2021) To React or Not to React: The Dilemma of Fish Immune Systems Facing Myxozoan Infections. Front Immunol 12:734238. 10.3389/fimmu.2021.734238 PMC8481699 34603313

[CR32] Hughes J. (2023). N SW Stock Status Summary 2021/22 – Yellowtail Kingfish (Seriola lalandi). NSW Department of Primary Industries - Fisheries, p 26. https://share.google/yROoB3h00jWNKJHzI

[CR33] Hughes GN, Streeting LM, Burns A, Mcdonald PG (2023) Towards a Diagnostic Tool for Turtle Ootaxonomy: Investigation of Microstructural Differences in the Eggshells of Australian Freshwater Turtles. Ichthyol Herpetology 111(1). 10.1643/h2021125

[CR34] Hutson K (2007) Parasite interactions between wild and farmed Yellowtail Kingfish (Seriola lalandi) in southern Australia. PhD Thesis. The University of Adelaide. https://share.google/T7geMS34hM7iSm1we

[CR35] Hutson K, Ernst I, Mooney AJ, Whittington ID (2007a) Metazoan parasite assemblages of wild Seriola lalandi (Carangidae) from eastern and southern Australia. Parasitol Int 56(2):95–105. 10.1016/j.parint.2006.12.003 17267264

[CR36] Hutson K, Ernst I, Whittington ID (2007b) Risk assessment for metazoan parasites of yellowtail kingfish Seriola lalandi (Perciformes: Carangidae) in South Australian sea-cage aquaculture. Aquaculture 271(1):85–99. 10.1016/j.aquaculture.2007.03.020

[CR37] Hutson KS, Davidson IC, Bennett J, Poulin R, Cahill PL (2023) Assigning cause for emerging diseases of aquatic organisms. Trends Microbiol 31(7):681–691. 10.1016/j.tim.2023.01.012 36841735

[CR38] Ishimaru K, Matsuura T, Tsunemoto K, Shirakashi S (2014) Seasonal monitoring of Kudoa yasunagai from sea water and aquaculture water using quantitative PCR. Dis Aquat Organ 108(1):45–52. 10.3354/dao02702 24492053

[CR39] Iwashita Y, Kamijo Y, Nakahashi S, Shindo A, Yokoyama K, Yamamoto A, Omori Y, Ishikura K, Fujioka M, Hatada T, Takeda T, Maruyama K, Imai H (2013) Food Poisoning Associated with Kudoa Septempunctata. J Emerg Med 44(5):943–945. 10.1016/j.jemermed.2012.11.026 23357380

[CR40] Jinnai M, Kawai T, Harada T, Nishiyama Y, Yokoyama H, Shirakashi S, Sato H, Sakata J, Kumeda Y, Fukuda Y, Ogata K, Kawatsu K (2017) Production of a novel monoclonal antibody applicable for an immunochromatographic assay for Kudoa septempunctata spores contaminating the raw olive flounder (Paralichthys olivaceus). Int J Food Microbiol 259:59–67. 10.1016/j.ijfoodmicro.2017.08.003 28822890

[CR41] Kawai T, Sekizuka T, Yahata Y, Kuroda M, Kumeda Y, Iijima Y, Kamata Y, Sugita-Konishi Y, Ohnishi T (2012) Identification of Kudoa septempunctata as the Causative Agent of Novel Food Poisoning Outbreaks in Japan by Consumption of Paralichthys olivaceus in Raw Fish. Clin Infect Dis 54(8):1046–1052. 10.1093/cid/cir1040 22281845

[CR42] Kleindienst RJ (2014) Investigating the effects of myxozoan parasites on post-harvest quality of Yellowtail Kingfish Seriola lalandi. Masters Thesis. Curtin University. https://share.google/ewcOzIapPStCbUc6U

[CR43] Koch R (1884) The etiology of tuberculosis. Mittheilungen aus dem Kaiserlichen Gesundheitsamte 2:1–88

[CR44] Kodo Y, Murata R, Suzuki J, Mori K, Chiba T (2026) Molecular detection of myxosporean parasites in human fecal samples associated with foodborne illness. Int J Food Microbiol 457:111842. 10.1016/j.ijfoodmicro.2026.111842 42119175

[CR45] Lauringson M, Kahar S, Veevo T, Silm M, Philpott D, Svirgsden R, Rohtla M, Päkk P, Gross R, Kaart T (2023) Spatial and intra-host distribution of myxozoan parasite Tetracapsuloides bryosalmonae among Baltic sea trout (Salmo trutta). J Fish Dis 46(10):1073–1083 10.1111/jfd.13827 37387198

[CR46] Lester RJG (1982) Unicapsula seriolae n. sp. (Myxosporea, Multivalvulida) from Australian Yellowtail Kingfish Seriola lalandi. J Protozoology 29(4):584–587. 10.1111/j.1550-7408.1982.tb01340.x

[CR47] Li YC, Inoue K, Zhang JY, Sato H (2022) New records of three commercial fish hosts for two Unicapsula spp. and Kudoa megacapsula (Myxozoa: Myxosporea: Multivalvulida). Parasitol Res 121(11):3133–3145. 10.1007/s00436-022-07584-7 35852603

[CR48] Liu Y, Wang Z, Huang Y, Konno K, Tong T, Yu C, Zhu B, Dong X (2022) Characteristic stable structure of the myosin rod in dark muscle of yellowtail kingfish (*Seriola aureovittata*). Int J Food Sci Technol 57(12):7917–7928. 10.1111/ijfs.16146

[CR49] LTHSWG (2024) Minutes - Line & trap harvest strategy working group meeting 5. https://www.dpi.nsw.gov.au/__data/assets/pdf_file/0011/1563869/Meeting-5-minutes-Line-and-Trap-Harvest-Strategy-Working-Group.PDF

[CR50] Malan N, Roughan M, Kerry C (2021) The Rate of Coastal Temperature Rise Adjacent to a Warming Western Boundary Current is Nonuniform with Latitude. Geophys Res Lett 48(3). 10.1029/2020gl090751

[CR51] Manlove K, Wilber M, White L, Bastille-Rousseau G, Yang A, Gilbertson ML, Craft ME, Cross PC, Wittemyer G, Pepin KM (2022) Defining an epidemiological landscape that connects movement ecology to pathogen transmission and pace‐of‐life. Ecol Lett 25(8):1760–1782 10.1111/ele.14032 35791088

[CR52] Miller T (2024) Chapter 13: Myxozoa (Subphylum) [Concepts in Animal Parasitology]. Concepts Anim Parasitol. 10.32873/unl.dc.ciap013

[CR53] Miller TL, Adlard RD (2013) Unicapsula species (Myxosporea: Trilosporidae) of Australian marine fishes, including the description of Unicapsula andersenae n. sp. in five teleost families off Queensland, Australia. Parasitol Res 112(8):2945–2957. 10.1007/s00436-013-3467-3 23812600

[CR54] Miller PA, Fitch AJ, Gardner M, Hutson KS, Mair G (2011) Genetic population structure of Yellowtail Kingfish (Seriola lalandi) in temperate Australasian waters inferred from microsatellite markers and mitochondrial DNA. Aquaculture 319(3):328–336. 10.1016/j.aquaculture.2011.05.036

[CR55] Munday PL, Schunter C, Allan BJM, Nicol S, Parsons DM, Pether SMJ, Pope S, Ravasi T, Setiawan AN, Smith N, Domingos JA (2019) Testing the adaptive potential of yellowtail kingfish to ocean warming and acidification. Front Ecol Evol 7. 10.3389/fevo.2019.00253

[CR56] Nadler LE, Adamo SA, Hawley DM, Binning SA (2023) Mechanisms and consequences of infection-induced phenotypes. Funct Ecol 37(4):796–800. 10.1111/1365-2435.14309

[CR57] Ogawa K (2023) Parasites in the Skeletal Muscle of Amberjacks Seriola spp. in Japan: A Review. Fish Pathol 58(4):127–145. 10.3147/jsfp.58.127

[CR58] Ohnishi T, Tomaru A, Yoshinari T, Kamata Y, Sugita-Konishi Y (2016) Detection of Myxosporean Parasites in Unidentified Food-Borne Disease Associated with Consumption of Raw Fish. Japanese J Food Microbiol 33(3):150–154. 10.5803/jsfm.33.150

[CR59] Ohnishi T, Obara T, Arai S, Yoshinari T, Sugita-Konishi Y (2018) Quantitative Analysis of Unicapsula seriolae in Greater Amberjack Associated with Unidentified Food-Borne Disease. Food Hygiene Saf Sci (Shokuhin Eiseigaku Zasshi) 59(1):24–29. 10.3358/shokueishi.59.24 29743464

[CR60] Oliver ECJ, Holbrook NJ (2018) Variability and Long-Term Trends in the Shelf Circulation Off Eastern Tasmania. J Geophys Research: Oceans 123(10):7366–7381. 10.1029/2018JC013994

[CR61] Paduano C, Partridge GJ, Saunders BJ, Woolley L (2026) Demand Feeders Provide a Direct Palatability Comparison for Assessing Aquaculture Diets on Cultured Yellowtail Kingfish, Seriola lalandi. Aquac Res 2026(1):8795431. 10.1155/are/8795431

[CR62] Phillips LR, Malan N, Roughan M, Harcourt R, Jonsen I, Cox M, Brierley AS, Slip D, Wilkins A, Carroll G (2022) Coastal seascape variability in the intensifying East Australian Current Southern Extension. Front Mar Sci 9. 10.3389/fmars.2022.925123

[CR63] Pirozzi I, Booth MA (2009) The routine metabolic rate of mulloway (Argyrosomus japonicus: Sciaenidae) and yellowtail kingfish (Seriola lalandi: Carangidae) acclimated to six different temperatures. Comp Biochem Physiol A: Mol Integr Physiol 152(4):586–592. 10.1016/j.cbpa.2009.01.008 19256082

[CR64] Puhr R, Heinze G, Nold M, Lusa L, Geroldinger A (2017) Firth’s logistic regression with rare events: accurate effect estimates and predictions? Stat Med 36(14):2302–2317. 10.1002/sim.7273 28295456

[CR65] Ray RA, Bartholomew JL (2013) Estimation of transmission dynamics of the Ceratomyxa shasta actinospore to the salmonid host. Parasitology 140(7):907–916 10.1017/S0031182013000127 23506996

[CR66] Rigos G, Katharios P, Kogiannou D, Cascarano CM (2021) Infectious diseases and treatment solutions of farmed greater amberjack < i>Seriola dumerili with particular emphasis in Mediterranean region. Reviews Aquaculture 13(1):301–323. 10.1111/raq.12476

[CR67] Rohde K (1976) Marine Parasitology in Australia. Parasitol Today Australian Supplement 7(11–1):477–482 10.1016/0169-4758(86)90211-5 15462827

[CR68] Samaranayaka AGP, Ho TCW, Li-Chan ECY (2007) Correlation ofKudoaSpore Counts with Proteolytic Activity and Texture of Fish Mince from Pacific Hake (Merluccius productus). J Aquat Food Prod Technol 15(4):75–93. 10.1300/J030v15n04_06

[CR69] Schmidt-Posthaus H, Wahli T (2015) Host and environmental influences on development of disease. Myxozoan Evol, Ecol Dev 281–293

[CR70] Shamsi S (2021) The occurrence of Anisakis spp. in Australian waters: past, present, and future trends. Parasitol Res 120(9):3007–3033. 10.1007/s00436-021-07243-3 34341859

[CR71] Shamsi S, Barton DP (2024) Exploring the Potential Role of the Genus Kudoa (Myxosporea: Kudoidae) as an Emerging Seafood-Borne Parasite in Humans. Curr Clin Microbiol Rep 11(2):107–114. 10.1007/s40588-024-00220-1

[CR72] Shamsi S, Gasser RB, Beveridge I (2011) Mutation scanning-coupled sequencing of nuclear ribosomal DNA spacers as a tool for the specific identification of different Contracaecum (Nematoda: Anisakidae) larval types. Mol Cell Probes 25(1):13–18. 10.1016/j.mcp.2010.09.003 20933594

[CR73] Shamsi S, Steller E, Chen Y (2018) New and known zoonotic nematode larvae within selected fish species from Queensland waters in Australia. Int J Food Microbiol 272:73–82. 10.1016/j.ijfoodmicro.2018.03.007 29550686

[CR74] Shin SP, Shirakashi S (2017) Investigation of *Kudoa yasunagai* (Hsieh & Chen, 1984) infection kinetics in *Seriola lalandi* (Valenciennes, 1833) by quantitative PCR. J Fish Dis 40(2):287–291. 10.1111/jfd.12507 27260675

[CR75] Smith A, Pepperell J, Diplock J, Dixon P (1991) Study suggests NSW kingfish one stock. Aust Fish 3:34–36. https://cir.nii.ac.jp/crid/1571417125513834112

[CR76] Sobral F, Roughan M, Malan N, Li J (2024) Mean-state and seasonal variability in temperature structure and heat transport in the East Australian current system from a multi-decadal regional ocean Model. J Geophys Research: Oceans 129(9):e2023JC020438. 10.1029/2023JC020438

[CR77] State of New South Wales (2026) Recreational fishing. State of New South Wales, NSW department of primary industries. Retrieved 18/05/2026 from. https://www.dpi.nsw.gov.au/fishing/recreational

[CR78] Stephens FJ (2016) Common pathogens found in yellowtail kingfish Seriola lalandi during aquaculture in Australia. Microbiol Australia 37(3):132–134. 10.1071/ma16045

[CR79] Stephens F, Savage A (2010) Two mortality events in sea-caged yellowtail kingfish *Seriola lalandi* Valenciennes, 1833 (Nannopercidae) from Western Australia. Aust Vet J 88(10):414–416. 10.1111/j.1751-0813.2010.00625.x 20854300

[CR80] Stewart J, Stuart S, Hughes JM, Booth DJ (2026) Improved estimates of growth for wild Yellowtail Kingfish (Seriola lalandi) off eastern-Australia using multiple data sources. Front Fish Sci 4. 10.3389/frish.2026.1733032

[CR81] Stone DAJ, Bansemer MS, Salini MJ (2022) Dietary lipid and protein levels influence the growth and feed utilisation of large yellowtail kingfish (Seriola lalandi) at summer water temperatures. Aquaculture 560:738567. 10.1016/j.aquaculture.2022.738567

[CR82] Symonds J, Walker S, Pether S, Gublin Y, McQueen D, King A, Irvine G, Setiawan A, Forsythe J, Bruce M (2014) Developing yellowtail kingfish (*Seriola lalandi*) and hāpuku (*Polyprion oxygeneios*) for New Zealand aquaculture. N Z J Mar Freshwat Res 48(3):371–384. 10.1080/00288330.2014.930050

[CR83] Tadiri CP, Scott ME, Fussmann GF (2018) Microparasite dispersal in metapopulations: a boon or bane to the host population? Proc Royal Soc B: Biol Sci 285(1885):20181519 10.1098/rspb.2018.1519 PMC6125888 30158314

[CR84] Tomochi H, Li YC, Tran BT, Yanagida T, Sato H (2014) Three Unicapsula species (Myxosporea: Trilosporidae) of Asian marine fishes, including the description of Unicapsula setoensis n. sp. in the yellowfin goby (Acanthogobius flavimanus) from the Inland Sea of Japan. Parasitol Res 113(10):3807–3816. 10.1007/s00436-014-4048-9 25088472

[CR85] Tout J, Siboni N, Messer LF, Garren M, Stocker R, Webster NS, Ralph PJ, Seymour JR (2015) Increased seawater temperature increases the abundance and alters the structure of natural Vibrio populations associated with the coral Pocillopora damicornis. Front Microbiol 6. 10.3389/fmicb.2015.00432 PMC4435422 26042096

[CR86] Tsuyuki H (1982) The relationship between acid and neutral protease activities and the incidence of soft cooked texture in the muscle tissue of Pacific hake (Merluccius productus) infected with Kudoa paniformis and/or K. thyrsitis, and held for varying times under different pre-freeze chilled storage conditions. Canadian Tech Rep Fish Aquat Sci

[CR87] Whipps CM, Adlard RD, Bryant MS, Lester RJ, Findlay V, Kent ML (2003) First report of three Kudoa species from eastern Australia: Kudoa thyrsites from mahi mahi (Coryphaena hippurus), Kudoa amamiensis and Kudoa minithyrsites n. sp. from sweeper (Pempheris ypsilychnus). J Eukaryot Microbiol 50(3):215–219. 10.1111/j.1550-7408.2003.tb00120.x 12836879

[CR88] Whipps CM, Grossel G, Adlard RD, Yokoyama H, Bryant MS, Munday BL, Kent ML (2004) Phylogeny of the multivalvulidae (Myxozoa: Myxosporea) based on comparative ribosomal DNA sequence analysis. J Parasitol 90(3):618–622. 10.1645/GE-153R 15270109

[CR89] Whipps CM, McAllister CT, Lindsay KA (2021) Genetic Diversity of < i>Cystodiscus Species in Amphibians in the Southern United States. J Parasitol 107(6):912–922. 10.1645/21-73 34847223

[CR90] Yunus M, Yustinasari LR, Natalia D, Ghosh S, Sakuma K, Inoue K, Sato H (2021) First report of three multivalvulid species (Cnidaria: Myxozoa: Myxosporea) in commercial fishes from Java Sea, Indonesia, with records of Unicapsula pyramidata and two new Kudoa spp. Parasitol Res 120(3):861–876. 10.1007/s00436-020-07030-6 33511471

