# Supplementary material for: Not all infected fish become mushy: phenotypic expression underestimates Unicapsula seriolae infection in yellowtail kingfish (Seriola lalandi)
Source: Parasitol Res. 2026 Jul 28;125(1):132. doi: 10.1007/s00436-026-08735-w (PMC13690875; doi:10.1007/s00436-026-08735-w)
Supplement: Supplementary file 1 [file 436_2026_8735_MOESM1_ESM.docx]

**Supplementary information**

**Supplementary Table S1** Questions in mushy fish story map survey

| **Question number** | **Question and unit of measurement** |
| --- | --- |
| 1 | Location of where fish was caught (selected on map or by entering the latitude and longitude) |
| 2 | Species of fish |
| 3 | Length (cm) |
| 4 | Depth caught (m) |
| 5 | Weight (Kg) |
| 6 | Visual parasites or injuries present |
| 7 | Temperature of water (°C) |
| 8 | If cooked, method |
| 9 | Part of fish cooked |
| 10 | Other notes |
| 11 | Date caught |
| 12 | Time caught |
| 13 | Upload image |
| 14 | Any comments |

**Supplementary table S2** Descriptive statistics and univariable associations of PCR detection across categorical predictors, with unadjusted associations assessed using Fisher’s exact tests. Seasonal categories included low counts in several cells (e.g. autumn and unreported, n = 1) and should be interpreted cautiously. Mushy flesh condition showed a borderline, but not significant association with PCR detection (p = 0.064)

| **Predictor** | **Category** | **PCR negative** | **PCR positive** | **Total (*n*)** | **Fisher’s p-value** |
| --- | --- | --- | --- | --- | --- |
| **Bioregion** | Hawkesbury Shelf | 5 | 6 | 11 | 0.274 |
|  | Manning Shelf | 17 | 8 | 25 |  |
| **Season** | Autumn | 0 | 1 | 1 | 0.085 |
|  | Spring | 15 | 5 | 20 |  |
|  | Summer | 3 | 2 | 5 |  |
|  | Winter | 3 | 6 | 9 |  |
|  | Unreported | 1 | 0 | 1 |  |
| **Tissue condition** | Mushy | 1 | 4 | 5 | 0.064 |
|  | Not mushy | 21 | 10 | 31 |  |

**Supplementary table S3** Individual fish-level dataset and inclusion across analyses. Each row represents a single fish sample. Columns indicate inclusion in different analytical steps: Fisher’s exact tests (based on available categorical data), initial binomial GLMs, univariable Firth models (mushy only), and the multivariable Firth model (restricted to complete cases). Differences in inclusion reflect missing covariate data and explain variation in sample size across analyses.

| ID | Species | Bioregion | Season | Water temperature ('c) | Length (cm) | Mushy or not mushy | PCR result (0= undetected, 1= positive) | Included in fisher's exact test | Included in glm | Included in univariable firth | Included in multivariable firth |
| --- | --- | --- | --- | --- | --- | --- | --- | --- | --- | --- | --- |
| 1 | Yellowtail kingfish | Manning shelf | Winter | 19.2 | 72 | Not mushy | 0 | Yes | Yes | Yes | Yes |
| 2 | Yellowtail kingfish | Manning shelf | Winter | 19.2 | 72 | Not mushy | 1 | Yes | Yes | Yes | Yes |
| 3 | Yellowtail kingfish | Manning shelf | Autumn | 23.7 | 68 | Not mushy | 1 | Yes | Yes | Yes | Yes |
| 4 | Yellowtail kingfish | Manning shelf | Winter | 18.9 | 67 | Not mushy | 1 | Yes | Yes | Yes | Yes |
| 5 | Yellowtail kingfish | Manning shelf | Winter | 19.6 | 79.5 | Not mushy | 0 | Yes | Yes | Yes | Yes |
| 6 | Yellowtail kingfish | Manning shelf | Winter | 19.6 | 79.5 | Not mushy | 1 | Yes | Yes | Yes | Yes |
| 7 | Yellowtail kingfish | Manning shelf | Winter | 19.6 | 79.5 | Not mushy | 1 | Yes | Yes | Yes | Yes |
| 8 | Yellowtail kingfish | Manning shelf | Winter | 19.6 | 79.5 | Not mushy | 0 | Yes | Yes | Yes | Yes |
| 9 | Yellowtail kingfish | Manning shelf | Winter | 20 | 77 | Not mushy | 1 | Yes | Yes | Yes | Yes |
| 10 | Yellowtail kingfish | Manning shelf | Spring | 20.4 | 82 | Not mushy | 1 | Yes | Yes | Yes | Yes |
| 11 | Yellowtail kingfish | Manning shelf | Spring | 20.4 | 91 | Not mushy | 0 | Yes | Yes | Yes | Yes |
| 12 | Yellowtail kingfish | Manning shelf | Spring | 20.4 | 70 | Not mushy | 0 | Yes | Yes | Yes | Yes |
| 13 | Yellowtail kingfish | Manning shelf | Spring | 21.6 | 72 | Not mushy | 0 | Yes | Yes | Yes | Yes |
| 14 | Yellowtail kingfish | Manning shelf | Spring | 21.6 | 72 | Not mushy | 0 | Yes | Yes | Yes | Yes |
| 15 | Yellowtail kingfish | Manning shelf | Spring | 21.6 | 72 | Not mushy | 0 | Yes | Yes | Yes | Yes |
| 16 | Yellowtail kingfish | Manning shelf | Spring | 21.6 | 72 | Not mushy | 0 | Yes | Yes | Yes | Yes |
| 17 | Yellowtail kingfish | Manning shelf | Spring | 21.6 | 72 | Not mushy | 0 | Yes | Yes | Yes | Yes |
| 18 | Yellowtail kingfish | Hawkesbury shelf | Winter | 16 | 100 | Not mushy | 1 | Yes | Yes | Yes | Yes |
| 19 | Yellowtail kingfish | Hawkesbury shelf | Spring | 19 | 70 | Not mushy | 1 | Yes | Yes | Yes | Yes |
| 20 | Yellowtail kingfish | Hawkesbury shelf | Spring | 19 | 80 | Not mushy | 0 | Yes | Yes | Yes | Yes |
| 21 | Yellowtail kingfish | Hawkesbury shelf | Spring | 19 | 80 | Not mushy | 0 | Yes | Yes | Yes | Yes |
| 22 | Yellowtail kingfish | Hawkesbury shelf | Spring | 19 | 80 | Not mushy | 0 | Yes | Yes | Yes | Yes |
| 23 | Yellowtail kingfish | Hawkesbury shelf | Spring | 20 | 95 | Not mushy | 0 | Yes | Yes | Yes | Yes |
| 24 | Yellowtail kingfish | Hawkesbury shelf | Spring | 23 | 70 | Not mushy | 1 | Yes | Yes | Yes | Yes |
| 25 | Yellowtail kingfish | Hawkesbury shelf | Spring | 20 | 132 | Mushy | 1 | Yes | Yes | Yes | Yes |
| 26 | Yellowtail kingfish | Manning shelf | Spring | 21.9 | Na | Not mushy | 0 | Yes | No | Yes | No |
| 27 | Yellowtail kingfish | Hawkesbury shelf | Spring | 21.5 | 111 | Mushy | 1 | Yes | Yes | Yes | Yes |
| 28 | Yellowtail kingfish | Hawkesbury shelf | Summer | 22.4 | 110 | Mushy | 1 | Yes | Yes | Yes | Yes |
| 29 | Yellowtail kingfish | Manning shelf | Un-  reported | Na | Na | Mushy | 0 | Yes | No | Yes | No |
| 34 | Yellowtail kingfish | Manning shelf | Spring | 22.8 | Na | Not mushy | 0 | Yes | No | Yes | No |
| 35 | Yellowtail kingfish | Manning shelf | Spring | 23.3 | 68 | Not mushy | 0 | Yes | Yes | Yes | Yes |
| 36 | Yellowtail kingfish | Manning shelf | Summer | 25.6 | 90 | Not mushy | 0 | Yes | Yes | Yes | Yes |
| 37 | Yellowtail kingfish | Manning shelf | Summer | 25.6 | 90 | Not mushy | 0 | Yes | Yes | Yes | Yes |
| 38 | Yellowtail kingfish | Manning shelf | Summer | 25.6 | 90 | Not mushy | 0 | Yes | Yes | Yes | Yes |
| 39 | Yellowtail kingfish | Manning shelf | Summer | 26.1 | 120 | Mushy | 1 | Yes | Yes | Yes | Yes |
| 40 | Yellowtail kingfish | Hawkesbury shelf | Spring | 21.2 | 91 | Not mushy | 0 | Yes | Yes | Yes | Yes |

**Supplementary Table S4.** Initial binomial GLM diagnostic output for PCR detection. Initial binomial GLMs were fitted to assess model stability prior to applying penalised regression. (A) Model fit statistics and diagnostic summaries. (B) Full coefficient estimates from the maximum likelihood GLM. Inflated standard errors and extreme coefficient estimates indicate quasi-complete separation and model instability. These diagnostics motivated the use of Firth penalised logistic regression for primary inference.

**A) Model summary and diagnostics**

| **Metric** | **Value** |
| --- | --- |
| *n* | 33 |
| Null deviance | 44.99 (df = 32) |
| Residual deviance | 32.89 (df = 28) |
| AIC | 42.89 |
| Observations removed | 3 (missing co-variate data) |

**B)** **Coefficients (GLM, MLE)**

| **Predictor** | **Estimate** | **SE** | **z** | **p-value** |
| --- | --- | --- | --- | --- |
| Intercept | 36.99 | 2985.85 | 0.012 | 0.990 |
| Bioregion (Manning Shelf) | 0.042 | 1.051 | 0.040 | 0.968 |
| Water temperature | -0.449 | 0.287 | -1.565 | 0.118 |
| Length (midpoint corrected) | -0.067 | 0.061 | -1.102 | 0.270 |
| Mushy (Not mushy) | -23.26 | 2985.83 | -0.008 | 0.994 |

**Supplementary table S5** Univariable Firth logistic regression model for PCR detection across the mushy phenotype. Not mushy fish have ~84% lower odds of infection compared to mushy fish, but this is not significant at p=0.053.

| Predictor | Level | OR | 95% CI | p-value |
| --- | --- | --- | --- | --- |
| Mushy condition | Not Mushy (ref = Mushy) | 0.16 | 0.01–1.03 | 0.053 |

**Supplementary table S6**. Full Firth penalised logistic regression output. Coefficients are presented on the log-odds scale. Confidence intervals and p-values are based on profile penalised likelihood (method = 2). Overall model fit statistics were: likelihood ratio test χ² = 8.50 on 4 df, p = 0.075; Wald test χ² = 5.79 on 4 df, p = 0.216; n = 33. Full model output is provided for transparency, while adjusted odds ratios and confidence intervals are summarised in Table 2.

| **Predictor** | **Coefficient** | **SE** | **Lower 95% CI** | **Upper 95% CI** | **χ²** | **p-value** |
| --- | --- | --- | --- | --- | --- | --- |
| Intercept | 14.71 | 8.46 | -0.07 | 39.78 | 3.80 | 0.051 |
| Bioregion (Manning Shelf) | 0.048 | 0.908 | -1.79 | 2.00 | 0.003 | 0.959 |
| Water temperature | -0.312 | 0.213 | -0.88 | 0.083 | 2.29 | 0.130 |
| Length (midpoint corrected) | -0.045 | 0.047 | -0.164 | 0.047 | 0.90 | 0.344 |
| Mushy (Not mushy) | -5.343 | 2.687 | -13.32 | -0.687 | 5.32 | **0.021** |

**Supplementary Table S7**. Data entered by anglers into the ESRI Story Map between October 2024 and November 2025.

| **ID** | **Season** | **Species** | **Longitude** | **Latitude** | **Length (cm)** | **Weight (kg)** | **Depth caught (m)** | **Visual parasites or injuries present?** | **Water temperature (°C)** | **If cooked, method:** | **Part of fish cooked** | **Other notes** | **Comments** |
| --- | --- | --- | --- | --- | --- | --- | --- | --- | --- | --- | --- | --- | --- |
| 1 | Spring | Yellowtail Kingfish | 151.2671 | -33.9256 | 76 | 10 | 10 | Not Sure | 19 | Broiled | Fillets with skin |  |  |
| 2 | Spring | Yellowtail Kingfish | 153.1 | -30.9384 | 72 |  | 25 |  | 22.6 | Pan Fried | Fillets without skin | Same trip had caught another kingfish 9.5kg 101cms which was fine, I didn’t think you could identify infected fish until I cut the smaller infected one and a very slight change is flesh was noticeable, cooked a piece of both fish to find out if infection was present, smaller fish had the parasite so generally we keep those for sashimi which hasn’t caused any health problems eating the raw infected fish. | Finding kudoa more prominent in our area. Five years ago you would be lucky to get an infected fish. These days it’s 1 in 4 at the least I would say! Very disappointing to dispatch these fish unnecessarily. |
| 3 | Spring | Mahi Mahi | 152.734 | -31.6392 | 83 | 3.5 | 82 |  | 22.2 |  |  |  | Keen to help your research with 3 of my stories but the comments section doesn’t allow enough letters. Ring if you wish. |
| 4 |  | Kingfish | 0 | 0 |  |  |  |  |  |  |  |  | Kingfish go mushy cause of the warmer water. |
| 5 | Spring | Yellowtail Kingfish | 153.1523 | -26.8205 | 130 | 15 | 20 | Not Sure | 22 | Pan Fried | Fillets without skin |  |  |
| 6 |  | Yellowtail Kingfish | 153.2355 | -26.6415 | 112 | 13.5 | 22 | Not Sure | 24 | Pan Fried | Fillets without skin |  | We caught 3 kingfish that day in roughly the same area. Only 1 was mushy. We observe that roughly 1 in 3 kingfish in this area are mushy. Anecdotal evidence. |
| 7 | Summer | Kingfish | 151.2862 | -33.7985 | 93 | 6.8 | 10 | Not Sure | 21 | Pan Fried | Whole fish | All mushy when cooked |  |
| 8 | Spring | Yellowtail Kingfish | 151.5849 | -33.2755 | 111 | 14 | 12 |  | 20 | Pan Fried | Fillets with skin |  | I had the fillets in the fridge for 3 days before I froze them, would they be of use for analysis? Call on: |
| 9 | Winter | Kingfish | 153.1007 | -30.9394 | 72 |  |  | Not Sure | 19.2 | BBQ | Fillets without skin | Confirmed parasite with PCR | Second fish had the parasite confirmed by PCR, first fish from same site same day did not. |
| 10 | Winter | Kingfish | 153.101 | -30.9394 | 72 |  |  | Not Sure | 19.2 | BBQ | Fillets without skin | 1st fish of 2, this fish did not test positive for the parasite with PCR |  |
| 11 | Autumn | Kingfish | 153.0774 | -30.9504 | 68 |  |  | Not Sure | 23.7 | BBQ | Fillets without skin | Tested positive for the parasite with PCR |  |
| 12 | Winter | Kingfish | 153.0779 | -30.9496 | 67 |  |  | Not Sure |  |  |  | Tested positive for parasite with PCR |  |
| 13 | Winter | Kingfish x 4 individuals (68cm - 91cm) | 153.0781 | -30.9498 | 68 |  |  | Not Sure | 19.7 |  |  | 2 out of the 4 fish caught tested positive for the parasite using PCR, 2 did not test positive. |  |
| 14 | Winter | Kingfish | 153.1022 | -30.9396 | 77 |  |  | Not Sure | 20 |  |  | Tested positive for parasite with PCR |  |
| 15 | Spring | Kingfish | 153.1012 | -30.9389 | 82 |  |  | Not Sure | 20.4 |  |  | Tested positive for parasite with PCR |  |
| 16 |  | Kingfish | 153.1026 | -30.94 | 91 |  |  | Not Sure | 20.4 |  |  | Did not test positive for parasite using PCR |  |
| 17 |  | Kingfish | 153.1019 | -30.939 | 70 |  |  | Not Sure | 20.4 |  |  | Did not test positive for parasite using PCR |  |
| 18 |  | Kingfish (5 individuals 67–77cm each) | 153.1015 | -30.941 | 77 |  |  | Not Sure | 21.6 |  |  | None tested positive for the parasite using PCR |  |
| 19 | Winter | Kingfish | 153.7979 | -28.8536 | 95 | 10 | 100 | Not Sure | 22 | Pan Fried | Fillets without skin |  | Flesh was firm until cooked. |
| 20 | Spring | Kingfish | 151.3219 | -33.5949 | 132 | 22 | 7 | Not Sure | 20 | BBQ | Fillets without skin |  |  |
| 21 | Summer | Yellowtail Kingfish | 152.3143 | -32.663 | 80 | 3.5 | 40 | Not Sure | 22 | Pan Fried | Fillets without skin |  | It was disappointing and disgusting. |
| 22 | Summer | Yellowtail Kingfish | 114.8682 | -29.45 | 80 |  | 18 | Not Sure | 22 | Pan Fried | Fillets without skin |  | Melted in the pan. |
| 23 | Summer | Kingfish | 153.198 | -30.2957 | 110 | 10 | 40 | Not Sure | 23 | BBQ | Fillets with skin | Bought at fish auction | Have caught several kingfish and bream around Coffs Harbour with similar mushy flesh. |
| 24 | Summer | Long Tom | 152.7933 | -31.6514 | 200 | 0.15 | 1 | Both Parasites & Injuries | 20 |  |  | Just saw a Long Tom on the surface while swimming laps of Pilot Beach Dunbogan 2443 it was swimming weakly and looked to be falling apart. I wasn’t fishing. This is just my observation. | Hope this was helpful. |
| 25 | Winter | Yellowtail Kingfish | 153.47 | -27.0615 | 113 |  | 30 | Not Sure |  | Pan Fried | Fillets with skin |  | Two kingfish were caught that day. Both were caught in the same location and only one presented with mushy flesh after it was cooked. |
| 26 | Summer | Jewfish | 151.4072 | -33.6271 | 73 | 4 | 47 | Parasites | 23 | Pan Fried | Fillets with skin | Couldn’t release it | Caught in a structure, I do have the whole fish if needed…in freezer. |
| 27 | Summer | Yellowtail Kingfish | 151.6327 | -33.0513 | 80 | 6 | 5 |  | 23 | Pan Fried | Fillets without skin |  | Half of the kingfish caught in end Dec to mid Jan have been mushy when cooked. This is the first time we have experienced this which led to googling what was wrong. We will no longer fish for them as it is very off putting. Lake Macquarie NSW. |
| 28 | Summer | Yellowtail Kingfish | 151.3039 | -33.8361 | 91 | 9 | 27 | Not Sure | 23.7 | BBQ | Fillets without skin |  | Fish filleted as per normal and felt fine. Turned to mush when cooking. Bitter taste. |
| 29 | Summer | Mahi Mahi | 151.8057 | -34.3314 | 75 | 2.65 | 120 | Not Sure | 22.7 |  |  | Fish caught and dispatched immediately then gutted and placed on ice for 24 hours. This is my usual method. | I caught 3 mahi mahi around the same area. This was the smallest of the 3. The other fish measured at 1.0m (4.17kg gutted) and 1.2m forgot the weight. Neither of the two bigger fish were affected. The affected fish was not eaten. |
| 30 | Summer | Kingfish | 144.9266 | -38.3796 | 72 | 4 | 15 | Not Sure | 19.5 | Raw/Sashimi | Fillets without skin | Seamed fine |  |
| 31 | Summer | Yellow tail kingfish | 151.1224 | -33.9696 | 123 | 15 | 5 | Not Sure | 23 | Pan Fried | Fillets without skin |  | Absolutely fine as sashimi/ uncooked/ even ceviche. Once under heat, it became very mushy. Eaten once a day since caught, worst on day 6 and incredibly unpleasant :(. |
| 32 |  | Yellowfin Bream | 153.4649 | -28.9545 |  |  |  |  |  | Pan Fried | Whole fish | Fished once or twice at the southern abuttment of Wardell Bridge on Richmond River in December/January five to 10 years ago. One third to half of all the Yellowfin Bream caught and kept were mushy when cooked. Two Dusky Flathead caught with the Yellowfin Bream and eaten did not have any issues. | From memory bag limit of Yellowfin Bream kept ranged in length from say 26–33cm. |
| 33 | Summer | Kingfish | 0 | 0 | 65 | 3.5 | 7 | Not Sure | 18 | Pan Fried | Fillets with skin |  | These kingfish were caught in the 1950s in Western Port Bay Victoria. They were inedible as they turned to mush. They were so bad we stopped fishing for them. Kingfish caught off Lord Howe and Montague Islands are good. |
| 34 | Summer | Kingfish | 0 | 0 | 1200 | 15 | 6 |  | 22 | BBQ | Fillets without skin |  | Fish was speared at little gibber head land and looked to be in remarkable health, fish was bled and iced immediately. |
| 35 | Spring | Kingfish | 152.2876 | -32.6077 |  | 16.1 | 6 | Injuries |  | Pan Fried | Fillets without skin | Kingfish had what looked like a healed over puncture mark. | Mushy when cooked! |
| 36 | Summer | Mahi Mahi | 150.9663 | -34.5681 | 62 |  | 112 | Not Sure | 23.5 | Pan Fried | Fillets without skin | Caught, filleted and refrigerated, then eaten 2 days later |  |
| 37 | Autumn | Kingfish | 151.1838 | -33.8753 |  |  |  | Parasites |  | Broiled | Fillets without skin | We did not catch. We were at [a] Restaurant Barangaroo, NSW | We were at restaurant. We had one, sent back to kitchen. They came back ten mins later and said the same thing happened with a second kingfish. We had no idea this problem existed. |
| 38 | Autumn | Yellowtail Kingfish | 151.4516 | -33.449 | 70 |  | 30 | Not Sure | 24 | Baked | Fillets without skin | Also pan fried, both turned mashed potato consistency |  |
| 39 | Autumn | Yellowtail Kingfish | 0 | 0 | 1100 | 0 | 45 | Parasites | 0 | Pan Fried | Fillets without skin | Fish was mushy and foul tasting |  |
| 40 | Autumn | Yellowtail Kingfish | 116.7465 | -35.0347 | 95 |  | 15 | Not Sure | 22 | Pan Fried | Fillets without skin | Mushy flesh when cooked, no visual parasites |  |
| 41 | Winter | Yellowtail Kingfish | 0 | 0 | 83 | 4 | 9 | Not Sure | 16 | Pan Fried | Fillets with skin | Very healthy, high fat visceral fat content | I catch a lot of kingfish in Port Hacking. Never had a soft flesh one that I have caught. I remember buying one from the market in the early 1980s which was the same. We mentioned it to the vendor. I think we got our money back. |
| 42 | Winter | Kingfish | 152.5291 | -32.4802 | 1.1 | 13 | 18 |  | 19 | Pan Fried | Fillets without skin | Fish didn’t present any illness or injury, a perfect looking kingfish that fought hard |  |
| 43 | Spring | Acanthopagrus australis (Yellowfin Bream) | 151.306 | -33.6349 | 35 | 0.5 | 4 |  | 18 | Foil in Oven | Whole fish |  |  |
| 44 | Spring | Kingfish | 151.3338 | -33.7411 | 132 |  |  |  |  | Pan Fried | Fillets without skin |  |  |
| 45 | Spring | Kingfish | 151.2879 | -33.7865 | 125 | 20 | 10 | Not Sure | 18 | Baked | Fillets with skin | At first, I didn’t think I had a mushy one because it didn’t fully turn to liquid, but it’s definitely not firm like a healthy kingfish should be |  |
| 46 | Spring | Kingfish | 151.3222 | -33.7329 | 120 | 12 | 10 | Not Sure | 19 | Foil in Oven | Fillets without skin |  |  |
| 47 | Spring | Kingfish | 0 | 0 |  |  |  |  |  |  |  |  | Purchased kingfish at [a retailer] Syd Fish Market yesterday. Cooked it and was mushy. |
| 48 | Spring | Kingfish | 153.607 | -26.3076 | 130 | 20 | 80 |  | 23 | Pan Fried | Fillets without skin |  | Fish looked normal and fought hard. |
